# (Im)Perfect robustness and adaptation of metabolic networks subject to metabolic and gene-expression regulation: marrying control engineering with metabolic control analysis

**DOI:** 10.1186/1752-0509-7-131

**Published:** 2013-11-21

**Authors:** Fei He, Vincent Fromion, Hans V Westerhoff

**Affiliations:** 1 The Manchester Centre for Integrative Systems Biology, Manchester Interdisciplinary Biocentre, University of Manchester, Manchester M1 7DN, UK; 2 Unité Mathématique, Informatique et Génomes, Institut National Recherche Agronomique, UR1077, F-78350 Jouy-en-Josas, France; 3 Department of Automatic Control and Systems Engineering, The University of Sheffield, Sheffield S1 3JD, UK; 4 Department of Synthetic Systems Biology and Nuclear Organization, Swammerdam Institute for Life Sciences, University of Amsterdam, Science Park 904, NL-1098 XH Amsterdam, The Netherlands; 5 Department of Molecular Cell Physiology, Faculty of Earth and Life Sciences, VU University Amsterdam, De Boelelaan 1085, NL-1081 HV Amsterdam, The Netherlands

**Keywords:** Metabolic control analysis, Control engineering, Transcriptional regulation, Synthetic biology, Robustness

## Abstract

**Background:**

Metabolic control analysis (MCA) and supply–demand theory have led to appreciable understanding of the systems properties of metabolic networks that are subject exclusively to metabolic regulation. Supply–demand theory has not yet considered gene-expression regulation explicitly whilst a variant of MCA, i.e. Hierarchical Control Analysis (HCA), has done so. Existing analyses based on control engineering approaches have not been very explicit about whether metabolic or gene-expression regulation would be involved, but designed different ways in which regulation could be organized, with the potential of causing adaptation to be perfect.

**Results:**

This study integrates control engineering and classical MCA augmented with supply–demand theory and HCA. Because gene-expression regulation involves time integration, it is identified as a natural instantiation of the ‘integral control’ (or near integral control) known in control engineering. This study then focuses on robustness against and adaptation to perturbations of process activities in the network, which could result from environmental perturbations, mutations or slow noise. It is shown however that *this* type of ‘integral control’ should rarely be expected to lead to the ‘perfect adaptation’: although the gene-expression regulation increases the robustness of important metabolite concentrations, it rarely makes them infinitely robust. For perfect adaptation to occur, the protein degradation reactions should be zero order in the concentration of the protein, which may be rare biologically for cells growing steadily.

**Conclusions:**

A proposed new framework integrating the methodologies of control engineering and metabolic and hierarchical control analysis, improves the understanding of biological systems that are regulated both metabolically and by gene expression. In particular, the new approach enables one to address the issue whether the intracellular biochemical networks that have been and are being identified by genomics and systems biology, correspond to the ‘perfect’ regulatory structures designed by control engineering vis-à-vis optimal functions such as robustness. To the extent that they are not, the analyses suggest how they may become so and this in turn should facilitate synthetic biology and metabolic engineering.

## Background

With the development of quantitative functional genomics approaches, it has become possible to analyse the cellular adaptation of cell physiology to altered environmental conditions experimentally, by monitoring changes in fluxes, metabolites, proteins or mRNAs. Such adaptations tend to occur at multiple regulatory levels if not simultaneously, then subsequently, depending on the time scales of observation [1-3]. In principle, an adaptive change in the rate of an enzyme (or flux) can be mediated by changes in (i) the concentration of metabolites (e.g. substrates, products and effectors) with direct, cooperative and allosteric effects on the activity of the enzyme [4], (ii) changes in the concentration of the enzyme through gene-expression alterations, and (iii) covalent modification via signal transduction. The first is termed metabolic (or enzymatic) regulation. The second is known as gene-expression (mediated) regulation and the third as signal-transduction (mediated) regulation. Because of similar properties, the latter two types of regulation have been considered together under the term ‘hierarchical regulation’ [2,5,6]. Although in this paper only the former two types of adaptive changes will be discussed explicitly, because of the above-mentioned similarities, the third type is addressed implicitly. Until now, significant progress has been made on the modelling of genome-scale metabolic networks in microorganisms integrating metabolic and gene-expression regulation [7,8]. The steady-state properties of a number of representative metabolic regulatory mechanisms, such as end-product inhibition, have been investigated substantially both in terms of metabolic control analysis (MCA) [9,10] and by the supply–demand theory championed by Hofmeyr and Cornish-Bowden [11-13]. In order to take gene-expression regulation into account, hierarchical control analysis (HCA) [14,15] has been developed as an extension to MCA, but it has not yet been linked up with the supply–demand theory. Developing such a link would seem useful as in quantitative experimental studies gene-expression regulation turned out to be as important as metabolic regulation [1,2,5,16].

The adaptive changes of reaction rates through metabolic and genetic regulation are usually due to feedback and/or feed-forward mechanisms. In biology, there is a perception that evolutionary optimization has made these mechanisms perfect. If this were so, this would suggest that such mechanisms might be identical to ‘perfect’ regulatory mechanisms designed by control engineering [17]. Indeed, Csete and Doyle [18] have suggested that such a convergent evolution of engineering and biology may have occurred. In particular, they came with an integral control structure containing both an actuator unit (corresponding to an integrator) and a controller/sensor unit. They showed that this regulatory structure would lead to a phenomenon called perfect adaptation and then proposed that such structures should be common to biology. In systems biology contexts, several biochemical processes have been discussed in terms of their control system structures. For example, robust perfect adaptation in bacterial chemotaxis signalling system, in mammalian iron and calcium homeostasis, and in yeast osmoregulation, have been interpreted as integral feedback control systems [19-22], without however proving that they corresponded precisely to the very same regulatory topology or even performance. A recent study identified the three different types of control structures used in control engineering, i.e. proportional, integral, and derivative control, in the regulation of energy metabolism [23]. With the exception of [22], the above work focused only on metabolic regulation, whereas [22] did not compare metabolic regulation with gene-expression regulation. In this study, the integration of metabolic and gene-expression regulation plus the integration between Metabolic Control Analysis and Control Engineering will be investigated.

Control engineering has examined which network structures may make adaptation of a network upon a sustained perturbation of a network component, ‘perfect’. Perfection was defined as the phenomenon that some important system variables (known as ‘controlled variables’) should be completely robust to the perturbations, i.e. with steady states values unaffected by the perturbations. Such perfect robustness can be achieved when a time integrator is applied to any variation of the controlled variable (or system error). This control feature is known as ‘integral control’. Through this time integral, the network would continue to change until the controlled variable is restored completely to its initial value. Because there must be some compensation for the perturbation, a different system variable then has to move away from its initial state. This so-called ‘manipulated variable’ is non-robust (fragile) to the perturbation, but enables the controlled variable to be robust.

If the control action is proportional to the variation of the perturbed variable itself, or a function thereof that is zero when that variation equals zero, the ultimate deviation of the controlled variable from its value before the perturbation, will be nonzero. This is the so-called ‘proportional control’ of control engineering. Perturbations may also result in a sustained oscillation of the controlled variable and to prevent this from happening, the third type of control focused on by control engineering can be useful, i.e. so-called ‘derivative control’ , which will not be discussed in this paper, but has been exemplified in reference [23].

As mentioned above, the mechanism of integral control is often referred to as ‘perfect adaptation’. Other authors have referred to similar network behaviour that was not based on the same integrative mechanism by the same phrase of perfect adaptation. One such case is that all steps in a metabolic pathway are regulated identically, i.e. their activities being modulated by the same factor. Tyson et al. [24] referred to this as perfect adaptation, but the mechanism hinges on precise regulation of various steps, we would suggest to refer to this as ‘perfect regulation’ since the adaptation part is not crucial. Kacser and Acerenza [25] called this the universal method for metabolic engineering. Fell and Thomas [26] proposed that this may be a common motif in biological regulation and Adamczyk et al. [27] elaborated it into the stealthy engineering principle. This paper will not discuss this perfect regulation mechanism, but focus on the robust perfect adaptation mechanism operating through integral control loops.

In this work, we shall try to bridge two rather unconnected approaches in analysing regulation of network properties. The one is that of control engineering which has devised networks structures that lead to perfect or imperfect adaptation. The other is that of biochemistry with MCA and supply–demand theory, as well as true-to-life examples of intracellular biochemical networks involving both metabolism and gene expression. We shall focus on pathways synthesizing precursors for macromolecule synthesis (proteins, nucleic acids) in which that precursor often inhibits an enzyme early in the pathway, both directly and through gene expression. Such end-product regulatory structures allow for some simplifications [28]. This makes them suitable for illustrating our relatively simple conclusions that are however valid more generally. We shall hypothesize that because a time integration of protein synthesis is involved, gene-expression regulation should be a prime example of integral control, whilst metabolic regulation is our candidate for the role of proportional control. We shall then interpret both these steady-state robustness properties and the control properties in terms of a new hierarchical supply–demand framework.

## Methods

### Kinetic description and classical control analysis

In this section, we demonstrate that the unique steady state of a metabolic network under regulations can be analysed by both the kinetics-based analysis and by metabolic (or hierarchical) control analysis. A hierarchical supply–demand theory linking hierarchical control analysis with classical supply–demand analysis, is developed for when gene-expression regulation is active. As a result the steady state properties of a metabolic network subject to various regulatory mechanisms can be analysed within a unified theoretical framework.

#### Basic regulatory architecture and kinetic analysis

**Figure 1:**
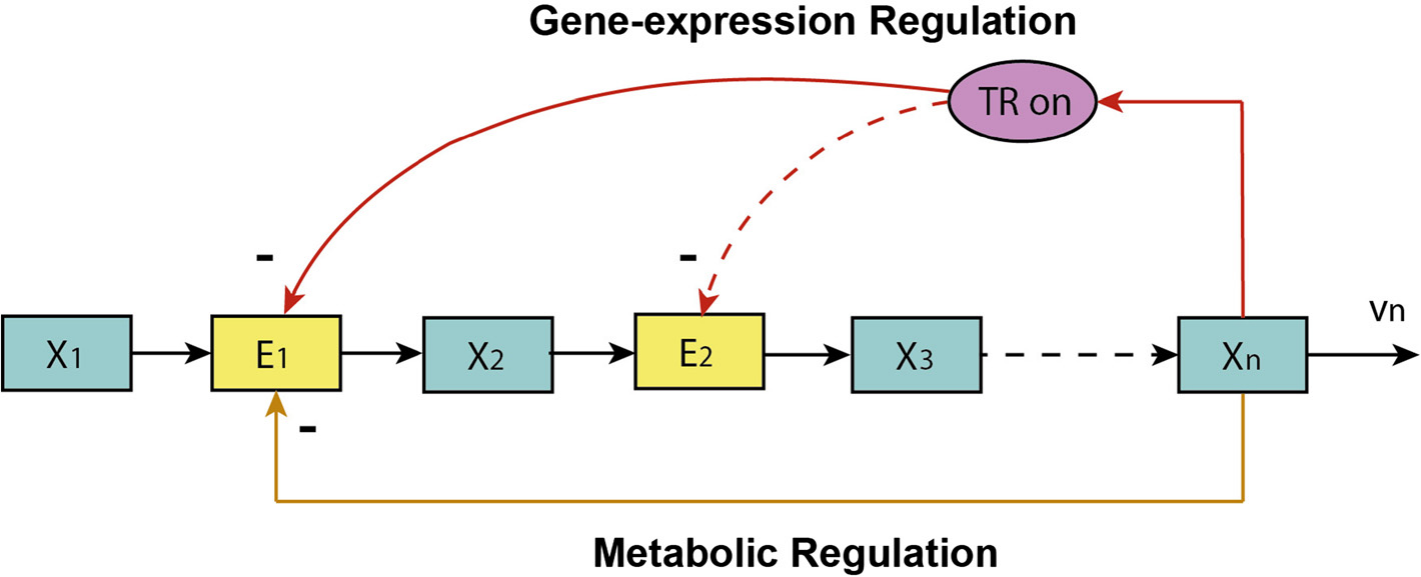
**The end-product module with gene-expression and metabolic regulation.***x*_1_, *x*_2_, …, *x*_*n*_ represent the concentrations of metabolites in the pathway; *E*_1_, *E*_2_, … are the concentrations of enzymes catalysing each reaction. For illustration, only the enzyme of the first reaction (*E*_1_) is assumed negatively regulated via both metabolic (allosteric) effect and gene-expression regulation through the end product *x*_*n*_.

The overall regulatory behaviour of a pathway can be decomposed into a number of elementary structures. Feedback inhibition by end-product has been reported for quite a few metabolic pathways, particularly in anabolism [29]. Another regulatory motif is the feed-forward activation of downstream enzymes (see Appendix A and [30]). In addition, in many metabolic pathways one or a few reactions are product insensitive. The reactions catalyzed by hexokinase, phosphofructokinase, and pyruvate kinase in mammalian glycolysis [31] and several steps in the central carbon metabolism in *B. subtilis*[28], constitute examples. The activities and concentrations of some of these enzymes are regulated by allosteric effectors, covalent modification or transcription. In this study we take a linear pathway with metabolic and gene-expression regulation of the first reaction through the end metabolite as the example of choice [28] (Figure 1). The first reaction (catalyzed by enzyme *E*_1_) is assumed to be insensitive to its immediate product. With this example we will be able to illustrate the essence of the principles we are after.

The following differential equations describe this end-product regulation pathway:

(1) $$\begin{array}{l} {\dot{x}}_{2}\left(t\right)=E_{1}\left(t\right)\cdot f_{1}\left(x_{1}\left(t\right),x_{n}\left(t\right),p\left(t\right)\right)-E_{2}\left(t\right)\cdot f_{2}\left(x_{2}\left(t\right),x_{3}\left(t\right)\right)\\ {\dot{x}}_{3}\left(t\right)=E_{2}\left(t\right)\cdot f_{2}\left(x_{2}\left(t\right),x_{3}\left(t\right)\right)-E_{3}\left(t\right)\cdot f_{3}\left(x_{3}\left(t\right),x_{4}\left(t\right)\right)\\ \vdots \;\vdots \;\vdots \\ {\dot{x}}_{n}\left(t\right)=E_{n-1}\left(t\right)\cdot f_{n-1}\left(x_{n-1}\left(t\right),x_{n}\left(t\right)\right)-E_{n}\left(t\right)\cdot f_{n}\left(x_{n}\left(t\right)\right)\\ {\dot{E}}_{1}\left(t\right)=g\left(x_{n}\left(t\right)\right)-k_{\mathrm{ED}}\cdot E_{1}\left(t\right) \end{array}$$

Here, we assume that the concentration of the substrate *x*_1_ is not influenced by the pathway and that only *E*_1_ is regulated through gene-expression. *x*_*i*_ is the concentration of *i*^th^ metabolite, and *f*_*i*_ describes the kinetics of the *i*^th^ reaction. Parameter *p* corresponds to other factors that could affect the activity of the first enzyme, e.g. co-factors or external metabolic modulators. Such effects on other enzymes are not addressed here. The gene expression function *g* is here assumed to depend on the ultimate metabolite only, the latter acting on the synthesis of the first enzyme. More realistic situations involving the dynamics of mRNA will be discussed in later sections. This paper is relevant for gene-expression regulation in general, i.e. includes regulation at the level of transcription, translation and post-translational modification, but our examples will mostly deal with only one type of these at a time and mainly consider transcription regulation. *k*_ED_ is the degradation rate constant of the first enzyme. In fast growing organisms and for stable proteins *k*_ED_ may merely represent the dilution effect due to cell growth and division (i.e. *k*_ED_ = *μ*, *μ* denoting the specific growth rate) [5,28], but in other cases it will depend on proteolysis, which will be discussed later.

**Figure 2:**
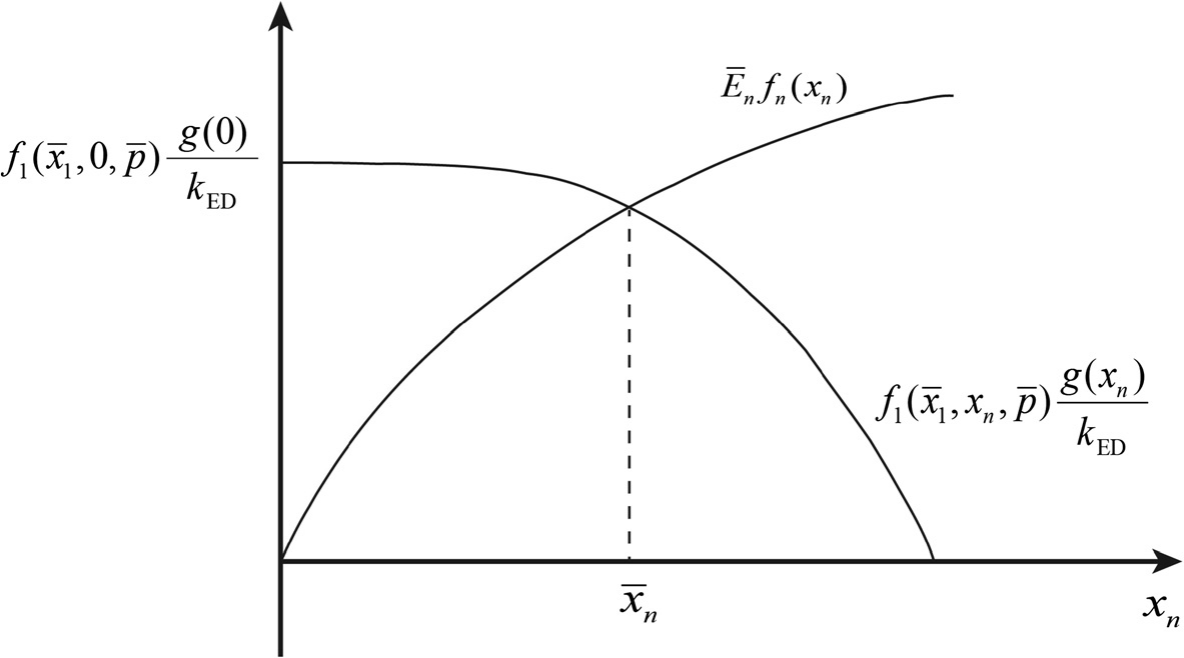
Illustration of the unique steady state regimen of a end-product module in terms of kinetics-based analysis.

By definition of the steady-state in living cells [32], *x*_1_(*t*) and *p*(*t*) are constants at steady state, i.e. $x_{1}\left(t\right)={\bar{x}}_{1}$ and $p\left(t\right)=\bar{p}$. Similarly, the concentrations of the enzymes are constants and equal to ${\bar{E}}_{2},\ldots ,{\bar{E}}_{n}$ respectively. Accordingly, if such a constant steady-state regime exists, and it is the unique solution of the following equation:

(2) $$f_{1}\left({\bar{x}}_{1},{\bar{x}}_{n},\bar{p}\right)\cdot \frac{g\left({\bar{x}}_{n}\right)}{k_{\mathrm{ED}}}={\bar{E}}_{n}\cdot f_{n}\left({\bar{x}}_{n}\right)$$

which only depends on the first and the end enzyme features and is only a function of the end product concentration *x*_*n*_. Usually, both $f_{1}\left({\bar{x}}_{1},x_{n},\bar{p}\right)$ and *g*(*x*_*n*_) are monotonically decreasing functions of *x*_*n*_, which describe the negative, metabolic and gene-expression regulation, respectively. Likewise, *f*_*n*_(*x*_*n*_) is usually a monotonically increasing function of *x*_*n*_. As shown by Equation (2), the steady-state regimen corresponds to the intersection of the two functions, and is unique due to the monotonic characteristics of *f*_1_, *f*_*n*_ and *g*, as illustrated in Figure 2.

Alternatively, if the *i*^th^ reaction (*i* > 1) is product insensitive but the first reaction is not, then *f*_1_ is re-defined and it also becomes a function of *x*_2_, while the kinetic function of the *i*^th^ step only depends on *x*_*i*_. It can be proven that at steady state, *x*_2_ then only depends on functions *f*_2_, …, *f*_*i*_ and is independent of *f*_*i* + 1_, …, *f*_*n* - 1_. This conclusion is both theoretically attractive and practically useful, because the steady state properties of an otherwise complex metabolic pathway may only depend on a limited number of enzyme features [33]. This is a case where the complexity of a pathway is limited; its flux and the concentrations of the upstream metabolites are only controlled by the properties of the upstream enzymes and the corresponding genes.

We will now examine how metabolic control analysis and supply–demand theory deal with these types of metabolic control structures.

#### Metabolic regulation: MCA and the supply–demand theory

**Figure 3:**
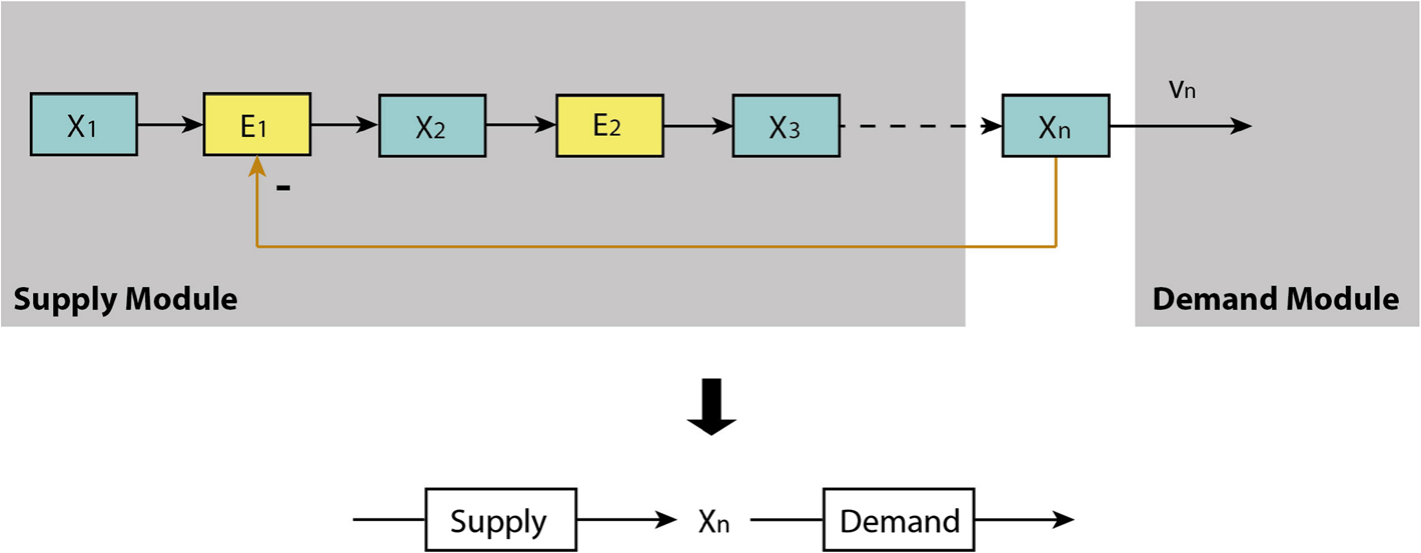
**The supply–demand structure of the end-product module with only metabolic regulation.** The upper diagram represents a linear pathway with only metabolic regulation from the end product to the first enzyme. The lower part is the supply–demand representation of the linear metabolic pathway.

Metabolic Control Analysis (MCA) has mostly dealt with the steady state properties of the metabolic part of schemas such as the ones mentioned in the previous subsection. Modular MCA [34] has divided metabolic networks of this type into modules with relatively autonomous activities, connected through well-identified metabolites. In the supply–demand theory, Hofmeyr and colleagues [12,13] have used a similar simplification to demonstrate much of the essence of the regulation of cell function. According to the latter, the metabolic part of the end-product pathway in Figure 1 can be partitioned into a two-step linear pathway with *supply* and *demand* blocks as shown in Figure 3.

The concentration and flux control coefficients of metabolic control analysis measure the steady state change in the concentration of a metabolite *X* and flux *J*, respectively, in response to a change in the activity of a process *i*. That change in activity may be effected by changing a parameter *p*_*i*_ (e.g. concentration of an inhibitor) that is specific for step *i*:

(3) $$\begin{array}{l} C_{i}^{X}=\left(\frac{\partial \ln X}{\partial \ln p_{i}}\right)/\left(\frac{\partial \ln v_{i}}{\partial \ln p_{i}}\right)=\frac{\partial \ln X}{\partial \ln v_{i}}\\ C_{i}^{J}=\left(\frac{\partial \ln J}{\partial \ln p_{i}}\right)/\left(\frac{\partial \ln v_{i}}{\partial \ln p_{i}}\right)=\frac{\partial \ln J}{\partial \ln v_{i}}\; \end{array}$$

The *v*_*i*_ is an activity rather than the rate of reaction *i* at steady state. When the parameter *p*_*i*_ is changed, *v*_*i*_ is not equal to the resultant change of the steady-state rate of reaction *i*; it is the change in that rate only if all the other variables that affect that rate been kept constants. The concentration control coefficients of the aforementioned supply–demand system can then be defined as $C_{s}^{x_{n}}=\partial \ln\left(x_{n}\right)/\partial \ln v_{\mathrm{supply}}$ and $C_{d}^{x_{n}}=\partial \ln\left(x_{n}\right)/\partial \ln v_{\mathrm{demand}}$, and the flux control coefficients, $C_{s}^{J}$ and $C_{d}^{J}$ similarly. These control coefficients describe the control exercised by a specific reaction or enzyme on the overall system variables or fluxes, while the ‘local’ regulatory properties of individual enzymes are quantified by the elasticity coefficients, such as

(4) $$\epsilon_{x_{n}}^{s}=\frac{\partial \ln v_{\mathrm{supply}}}{\partial \ln x_{n}},\;\epsilon_{x_{n}}^{d}=\frac{\partial \ln v_{\mathrm{demand}}}{\partial \ln x_{n}}$$

$\epsilon_{x_{n}}^{s}$ and $\epsilon_{x_{n}}^{d}$ measures how the reaction rates of the *supply* and *demand* blocks are influenced by the end product concentration *x*_*n*_. Because the rates are those of blocks rather than of single enzymes, these elasticities are named ‘overall elasticities’ [32]. They can be further expressed into more, though not quite elemental elasticity coefficients:

(5) $$\begin{array}{l} \epsilon_{x_{n}}^{s}=c_{1}^{J_{1}}\cdot \epsilon_{x_{n}}^{v_{1}}+c_{n-1}^{J_{1}}\cdot \epsilon_{x_{n}}^{v_{n-1}}\\ \epsilon_{x_{n}}^{d}=\epsilon_{x_{n}}^{v_{n}} \end{array}$$

where $\epsilon_{x_{n}}^{v_{1}}$ denotes the total elasticity of the first reaction with respect to *x*_*n*_ through the metabolic regulation. The lowercase flux control coefficients *c* now refer to the local control within the supply module only (if seen as if in isolation with *x*_*n*_ fixed). They can be obtained from summation and connectivity theorems as control coefficients of the module (see Appendix B). When assuming the first reaction is product insensitive, the *supply* modular elasticity in (5) can be further simplified as

(6) $$\epsilon_{x_{n}}^{s}=c_{1}^{J_{1}}\cdot \epsilon_{x_{n}}^{v_{1}}$$

In such a case, the first enzyme has the full control of flux (i.e. rate-limiting step) within the *supply* module, such that $c_{1}^{J_{1}}=1$ and

(7) $$\epsilon_{x_{n}}^{s}=\epsilon_{x_{n}}^{v_{1}}$$

Using the summation and connectivity theorems in MCA, all the four concentration and flux control coefficients can be expressed in terms of the elasticity coefficients (see Appendix B). For the concentration control of the *supply* step and the flux control of the *demand* step this becomes, using (5) and (7):

(8) $$\begin{array}{l} C_{s}^{x_{n}}=\frac{1}{\epsilon_{x_{n}}^{d}-\epsilon_{x_{n}}^{s}}=\frac{1}{\epsilon_{x_{n}}^{v_{n}}-\epsilon_{x_{n}}^{v_{1}}}\\ C_{d}^{J}=C_{v_{n}}^{J}=\frac{\epsilon_{x_{n}}^{s}}{\epsilon_{x_{n}}^{s}-\epsilon_{x_{n}}^{d}}=\frac{1}{1+\left|\epsilon_{x_{n}}^{d}/\epsilon_{x_{n}}^{s}\right|}=\frac{1}{1+\left|\epsilon_{x_{n}}^{v_{n}}/\epsilon_{x_{n}}^{v_{1}}\right|} \end{array}$$

These equations show that if the first reaction has complete flux control within the *supply* module, the control of steady state end-product concentration and flux are only functionally depend on two elasticity coefficients, i.e. the ones that correspond to the first and last reactions. When the feedback is very strong, i.e. $\left|\epsilon_{x_{n}}^{v_{1}}\right|\gg \left|\epsilon_{x_{n}}^{v_{n}}\right|$, the control of the demand flux $C_{d}^{J}$ is close to 1. The MCA and supply–demand simplification thereof discussed in this section partially explain the state-steady properties of the aforementioned full regulatory system, but only for the case of metabolic regulation. When including the gene-expression regulation, a more complete interpretation can be achieved by using the hierarchical control analysis and a new ‘hierarchical supply-demand’ theory as investigated in the next subsection.

#### Gene-expression and metabolic regulation: hierarchical supply–demand theory

Westerhoff and coworkers have developed hierarchical control analysis (HCA), an extension of MCA that can take gene-expression regulation and signal transduction into account [14,26]. We shall here implement this by extending the meaning of the elasticity coefficients in the previous subsection to include regulation through gene expressions.

**Figure 4:**
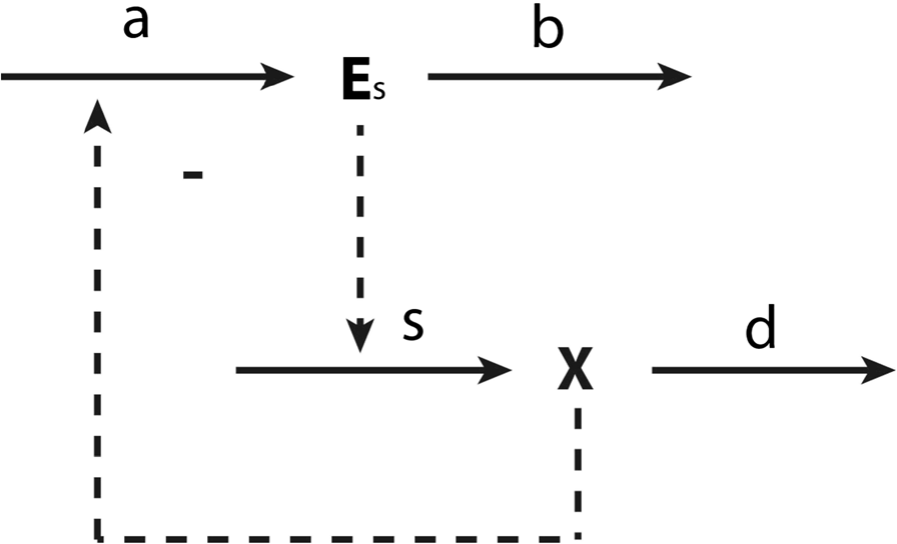
**Illustration of hierarchical control.** The lower part represents a metabolic supply–demand system, in which the supply is catalyzed by enzyme *E*_s_ (or enzymes stemming from an operon). The upper part describes the synthesis of enzyme *E*_s_ in process a and its degradation in process b. Full arrows represent chemical conversions. Dashed arrows represent allosteric influences or catalysis. ‘s’ and ‘d’ stand for ‘supply’ and ‘demand’, respectively.

When considering the metabolic part of the network alone, i.e. if gene expression were always the same, the control on the concentration of intermediate *X* in a supply–demand system follows (8). If the roles of the synthesis and degradation of metabolic enzymes are considered explicitly, as illustrated in Figure 4, HCA has to be introduced, and the corresponding hierarchical control coefficient becomes:

(9) $$H_{s}^{X}=\frac{\partial \ln X}{\partial \ln v_{\mathrm{supply}}}=\frac{1}{\epsilon_{X}^{d}-*\epsilon_{X}^{s}}$$

Capital *H* is here used for the hierarchical control coefficients as defined in Table 1. $*\epsilon_{X}^{s}$ is an “overall” elasticity coefficient, including a classical ‘direct elasticity’ only related with metabolic responses (i.e. $\epsilon_{X}^{s}$ similar to the $\epsilon_{x_{n}}^{s}$ defined in the MCA in (8)) and an ‘indirect elasticity’ due to gene-expression regulation:

**Table 1:** The list of symbols and definitions

**Symbol**	**Definition & comments**
$C_{i}^{f}$ or $c_{i}^{f}$	Metabolic control coefficients as defined in (3). *f* is the system function of interest (i.e. a particular flux, *J*_*j*_, or a metabolite concentration *X*). Lowercase *c* is used to represent control within a local network (e.g. supply module). The index *i* refers the process that is controlling.
$H_{i}^{f}$	Hierarchical control coefficient. Its mathematical definition is in the same form as metabolic control coefficients in (3), but the system under study can be more general. MCA only studies the control in a metabolic pathway or a signal transduction cascade, but not their combination. HCA investigates the control in a hierarchical regulatory network with interactions at different levels, i.e. metabolic, signal transduction, and gene-expression.
${ℜ}_{i}^{f}$	MCA (or HCA) based robustness coefficient.
	$${ℜ}_{i}^{f}\equiv \frac{1}{\left(\frac{\partial \ln f}{\partial \ln e_{i}}\right)}\equiv \frac{1}{C_{i}^{f}}\;\mathrm{or}\;\frac{1}{H_{i}^{f}}$$ .
$$F_{i}^{f}$$	Fragility coefficient. It is the inverse of the robustness coefficient and identical to the control coefficient. $F_{i}^{f}\equiv \frac{\partial \ln f}{\partial \ln e_{i}}\equiv \frac{1}{{ℜ}_{i}^{f}}\equiv C_{i}^{f}\;\mathrm{or}\;H_{i}^{f}.$
$\epsilon_{x_{i}}^{v_{j}}$	Elasticity coefficient defined in MCA. It denotes the immediate influences of metabolite *x*_*i*_ with respect to the reaction rate in the *j*^th^ step in the pre-steady state.
$\epsilon_{x_{i}}^{s}$ or $\epsilon_{x_{i}}^{d}$	Elasticity coefficient of metabolite *x*_*i*_ with respect to the metabolic supply (*s*) or demand (*d*) module, as defined in (4).
$*\epsilon_{x_{i}}^{v_{j}}$, $*\epsilon_{x_{i}}^{s}$ (or $*\epsilon_{x_{i}}^{d}$)	The overall elasticity (see [32]) for a reaction step under both metabolic and gene expression regulation, or the overall elasticity in a hierarchical supply or demand module.
*v*_Trsc_ or *v*_Trnl_	Reaction rate of transcription or translation
*v*_RD_ or *v*_ED_	Reaction rate of mRNA or protein degradation
*k*_RD_ or *k*_ED_	mRNA or protein degradation rate constant
$k_{\text{proteolysis}}^{i}$	Rate constant of *i*^th^ order proteolysis
*μ*	The cells’ specific growth (division) rate
*E*	Concentration of enzyme
*X or x*_*i*_	Concentration of intermediate metabolite
*R*	Concentration of mRNA
*r*	Reference signal.

(10) ϵXs*=ϵXs+ϵEss⋅caEs⋅ϵXa

The lower case *c* is used for ‘metabolic control coefficients’ , i.e. control coefficients that only take the local network (metabolic, or gene expression but not their combination) into account. $\epsilon_{E_{s}}^{s}$ is often equal to 1, i.e. when the rate of the reaction in isolation is proportional to the concentration of the enzyme catalyzing it.

Using metabolic control analysis for the gene expression part of the network, the control coefficient of the protein synthesis reaction with respect to the concentration of the protein synthesized is:

(11) $$c_{a}^{E_{s}}=\frac{1}{\epsilon_{E_{s}}^{b}-\epsilon_{E_{s}}^{a}}$$

Combining the above expressions, one can express the hierarchical coefficient quantifying the control exerted by the supply enzyme on the concentration of the metabolic intermediate *X* in terms of all the elasticity coefficients in the network:

(12) $$H_{s}^{X}=\frac{1}{\epsilon_{X}^{d}-\epsilon_{X}^{s}-\frac{\epsilon_{E_{s}}^{s}\cdot \epsilon_{X}^{a}}{\epsilon_{E_{s}}^{b}-\epsilon_{E_{s}}^{a}}}=\frac{1}{\epsilon_{X}^{d}+\left(-\epsilon_{X}^{s}\right)+\frac{\epsilon_{E_{s}}^{s}\cdot \left(-\epsilon_{X}^{a}\right)}{\epsilon_{E_{s}}^{b}+\left(-\epsilon_{E_{s}}^{a}\right)}}=-H_{d}^{X}$$

The terms in parentheses are usually positive. The equation shows that the control by supply (i) decreases with the absolute magnitudes of the elasticities with respect to *X* of the supply, of the demand, and of the protein synthesis, but (ii) increases for increasing elasticities of the protein synthesis and degradation reactions with respect to the concentration of the enzyme. The equation also shows that for finite non-zero magnitudes of the elasticities, the hierarchical control coefficients for control by supply may be decreased by elasticities in the gene-expression network, but is usually not brought down all the way to zero. The same applies to the control by demand, which is equal to minus the control by supply.

**Figure 5:**
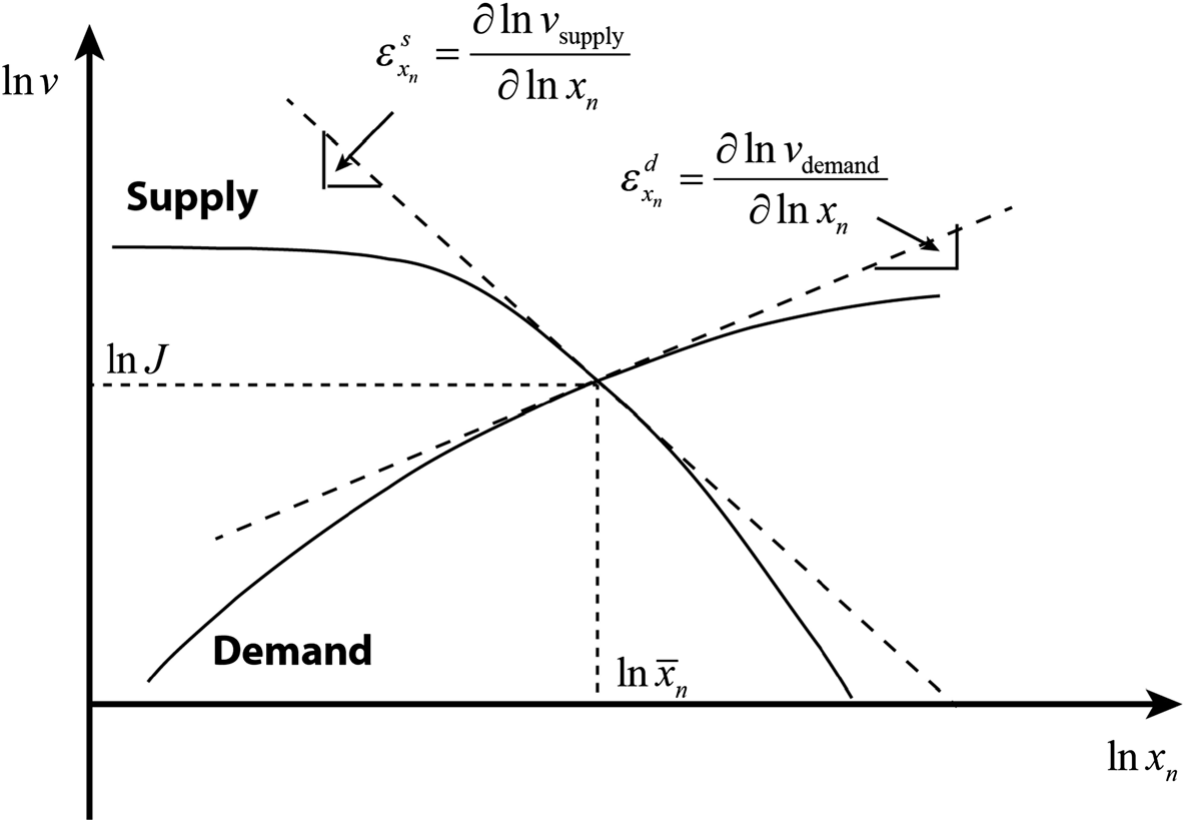
Illustration of the steady state properties of a supply-demand system in terms of changes in the flux, intermediate concentration and elasticity coefficients.

Now let us recall the end-product module with both metabolic and gene-expression regulatory feedbacks of Figure 1. As the end product (*x*_*n*_) regulates the first reaction through both metabolic regulation and gene-expression regulation, the corresponding ‘overall’ elasticity (see Table 1) of the first reaction can be expressed as:

(13) $$*\epsilon_{x_{n}}^{v_{1}}=\epsilon_{x_{n}}^{v_{1}}+\epsilon_{E_{1}}^{v_{1}}\cdot c_{v_{\mathrm{Trans}}}^{E_{1}}\cdot \epsilon_{x_{n}}^{v_{\mathrm{Trans}}}$$

where $\epsilon_{x_{n}}^{v_{1}}$ denotes the elasticity through metabolic regulation, $c_{v_{\mathrm{Trans}}}^{E_{1}}$ denotes the control of gene expression (i.e. transcription and translation) on the concentration of the first enzyme [15,35]. By replacing the $\epsilon_{x_{n}}^{v_{1}}$ in (7) with the more complete expression (13), i.e. using $\epsilon_{x_{n}}^{s}=*\epsilon_{xn}^{v1}$, the hierarchical control coefficient $H_{s}^{x_{n}}$ can be expressed into elasticity coefficients, in a similar manner as (12). Since both metabolic and gene-expression regulation constitute negative feedbacks, $\epsilon_{x_{n}}^{s}$ is negative and becomes more negative with increasing *x*_*n*_ due to increasing product inhibition. $\epsilon_{x_{n}}^{d}$ is positive and decreases asymptotically to zero with increasing *x*_*n*_. Therefore, the relationship between the reaction rate and end product concentration can be described in terms of the elasticities as depicted in Figure 5. Figure 5 explains the unique steady-state results obtained from classical steady state analysis (see Figure 2).

An illustration of the *supply–demand* relationship similar to Figure 5 has been presented in [13]. However, here we extend the interpretation of the system and corresponding elasticity coefficients to the more general case that includes gene-expression regulation. As an extension to the classical supply–demand theory, the analysis given in this section can be named the ‘hierarchical supply-demand’ theory.

### Control engineering

The discipline of Control Engineering first identifies a so-called *controlled variable*, which it sees as the output of the system. In metabolic biochemistry, output often relates to a flux, but can also be the concentration of an important metabolite in the pathway. Control engineering next examines the various categories of mechanism that may contribute to the capability of the network of maintaining the controlled variable close to its original steady state value when the system is subject to a sustained perturbation. The ‘error (function)’ is the deviation (*δX*) of the value of the controlled variable (*X*) from its value before the perturbation, or the difference to a reference signal *r*. The network ‘adapts’ to the perturbation of the controlled variable, i.e. to the error function, in a so-called ‘control action’. RNA polymerase plus the ribosomes that together translate changes in the concentration of metabolites to changes in gene expression, or direct metabolic regulation of the activity of an enzyme correspond to such control actions. The output of the control action (or of the ‘controller’) is often named the *manipulated variable*. In metabolism, reaction rates *v*(*X*, *E*) are variables manipulated either by the concentration of metabolites (*f*(*X*)) or by the concentration of the enzyme that catalyses the reaction concentration (*E*). A mechanical control system often includes an *actuator* that converts the control signal into some kind of mechanical motion. For a biochemical system discussed in this study, this may correspond to the enzyme catalysing the reaction synthesizing or degrading *X*. Usually there exists a *sensor* measures the controlled variable and translates its error function into the input signal of the controller. In a gene-expression regulation, the transcription factor can be regarded as the sensor.

The three most widely used categories of control are the proportional, integral, and derivative (PID) control mechanisms [17]. They differ depending on whether the control system’s response is a function of the ‘error function’ itself, the time integral thereof or the time derivative thereof, respectively. In systems biology literature, the proportional control mechanism has already been referred to in terms of metabolic regulation [19,20,23] (e.g. feedback inhibition). However, when Control Engineering discusses proportional control mechanisms, response is *proportional* to the error function. In actual biochemistry, enzyme *activity* is rarely a linear function of the concentrations of metabolites *X*, which includes the enzyme’s substrate, its product and allosteric modifiers. MCA accommodates this nonlinearity by allowing the elasticity coefficient to differ from 1. Metabolic regulation by the ‘error function’, is part of the nonlinear dynamics of the process or system, i.e. *f*(*X*), both conceptually and in the mathematical modelling. It would seem therefore that the proportional control of Control Engineering can be nonlinear in biochemical networks.

Integral control action through the accumulation of molecules in the metabolic process has also been reported [19,20,23]. Here the systems response should be a function not of the error function itself but of the integral of that error function. In the present study we examine gene-expression regulation from this point of view, since protein synthesis requires time integration and depend on the error function, and because changes in protein concentration directly affect the rate of the reaction the protein may catalyzes. We may expect that this integral control somehow corresponds to the ‘indirect elasticities’ of HCA. Whether indeed gene-expression regulation corresponds to an exact (or ideal) integral control mechanism will be further discussed in the Results and Discussion section. Whether there exists a derivative control action and whether it relates to a specific type of regulation in a metabolic pathway will not be investigated in this paper. Reference [23] already identified an example.

Considering the dynamics of a metabolic system, we can write the time dependence of the concentrations of its metabolites as:

(14) $$\dot{X}=N\cdot v\left(X,E\right)=N\cdot E\cdot f\left(X\right)$$

**Figure 6:**
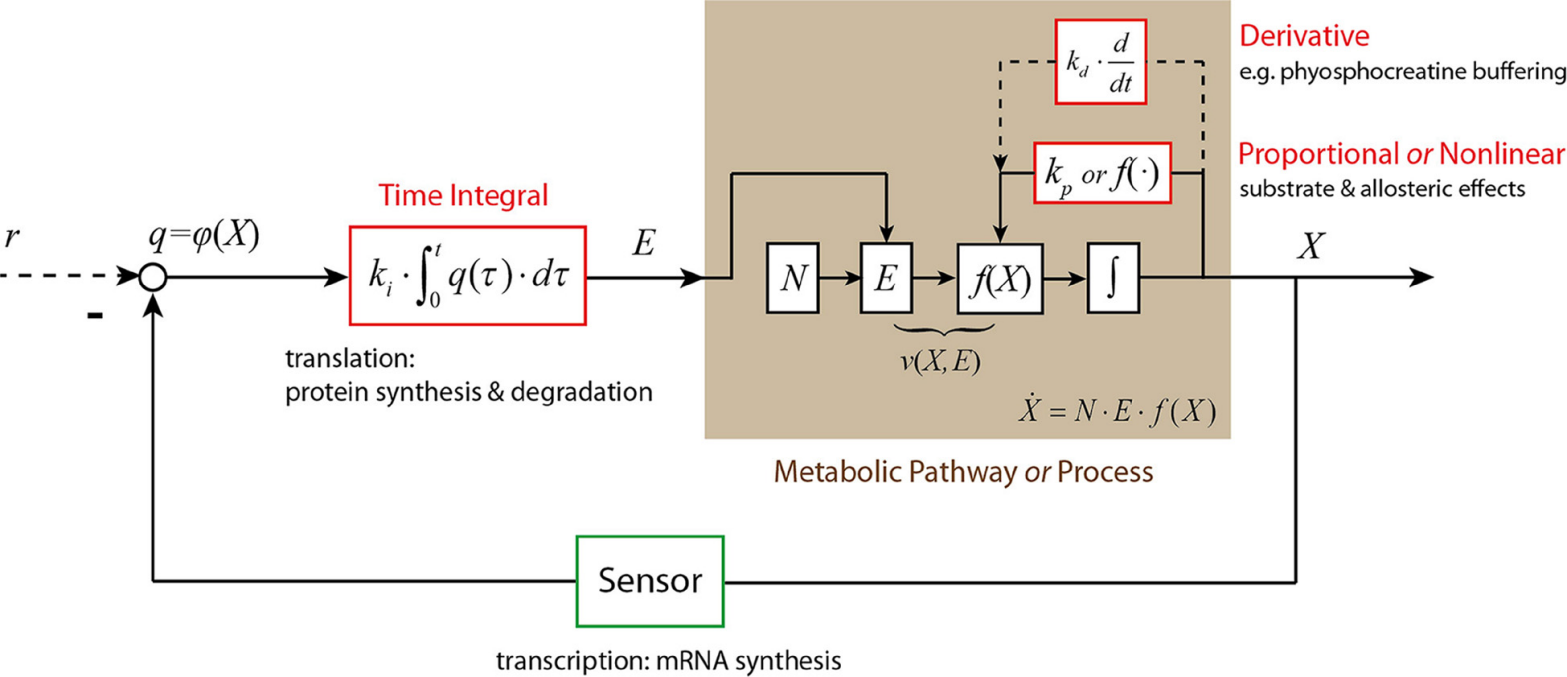
**The closed-loop control structure of a metabolic pathway subject to various types of regulation.** There are three types of feedback control mechanisms: i) proportional or nonlinear control that is related to the substrate or allosteric effects; ii) derivative control that can be related with signalling, e.g. phyosphocreatine buffering; and iii) integral control introduced by gene-expression regulation because protein synthesis requires time integration. The former two control loops i) and ii) are often modelled together with the dynamics of the metabolic process. Here, metabolite concentration *X* is the output of a metabolic process and is the *controlled variable* of different control loops. Reaction rates *v*(*X*,*E*) are the *manipulated variables* because they are functions of both enzyme concentration *E*, i.e. the output of integral control loop (gene-expression regulation), and metabolic process (*f*(*X*)), i.e. the outputs of proportional/nonlinear and derivative control loops (metabolic regulation or signalling). Here, the integral control input *q*(*t*) is a function of the controlled variable (*q*(*t*) = *φ*(*X*(*t*)) or the difference (or error) between the controlled variable and a reference signal (*r*).

*N* is the stoichiometry matrix. *E* is a diagonal matrix with the concentrations of the enzymes that catalyse the various reactions along its diagonal. *f*(*X*) is a vector function of the concentrations of the metabolites and kinetic parameter values. The regulated metabolic pathway can be described in terms of a closed-loop feedback control system, as indicated in Figure 6, in which *k*_*p*_, *k*_*i*_ and *k*_*d*_ are the PID control parameters. We here note that Figure 6 and subsequent figures refrain from biochemical detail. This is because the analysis in the present paper aims at obtaining a set of conclusions with general significance. Being specific in the schemes we use as illustrations would detract from this aim.

When considering a sustained perturbation *γ∙δp* (e.g. change in a parameter *p*) and denoting by *δ* the (small) deviation from the steady state prior to this perturbation, the time dependent variation in the metabolite concentrations may be observed:

(15) $$\delta \dot{X}=N\cdot \mathrm{\delta v}\left(X,E\right)=N\cdot E_{\mathrm{ss}}\cdot f^{\prime }\left(X\right)\cdot \mathrm{\delta X}+N\cdot f{\left(X\right)}_{\mathrm{ss}}\cdot \mathrm{\delta E}+\gamma \cdot \mathrm{\delta p}$$

The subscript *ss* refers to the steady state values. By substituting the time integration of gene expression, i.e. $k_{i}\cdot \int_{0}^{t}q\left(\tau \right)\cdot \mathrm{d\tau}$, for *δE*, and assuming the proportional and derivative actions a part of the metabolic process (14),

(16) $$\delta \dot{X}=N\cdot E_{\mathrm{ss}}\cdot f^{\prime }\left(X\right)\cdot \mathrm{\delta X}+N\cdot f{\left(X\right)}_{\mathrm{ss}}\cdot k_{i}\cdot \int_{0}\delta \varphi \left(X\right)\cdot \mathrm{dt}+\gamma \cdot \mathrm{\delta p}$$

This describes the overall dynamics of a closed-loop metabolic system under perturbation as a sum of three terms. The first term of these is a nonlinear function of (or in first order proportional to) the perturbation of the controlled variable (i.e. the error function) *δX*. This term describes all the direct elasticities, including non-regulatory system kinetics such as substrate and product effects, and (other) metabolic regulation such as allosteric activation. The second term corresponds to a time integral of a function of the perturbation of the controlled variable *δX*, and can also depend on other system variables as discussed in the Results and Discussion section.

We shall now examine whether these two terms in the equation (16), correspond to the proportional and integral control loops of Control Engineering.

## Results and discussion

### A simple example of combined metabolic and gene-expression regulation of an important intracellular process: ATP (energy) metabolism

**Figure 7:**
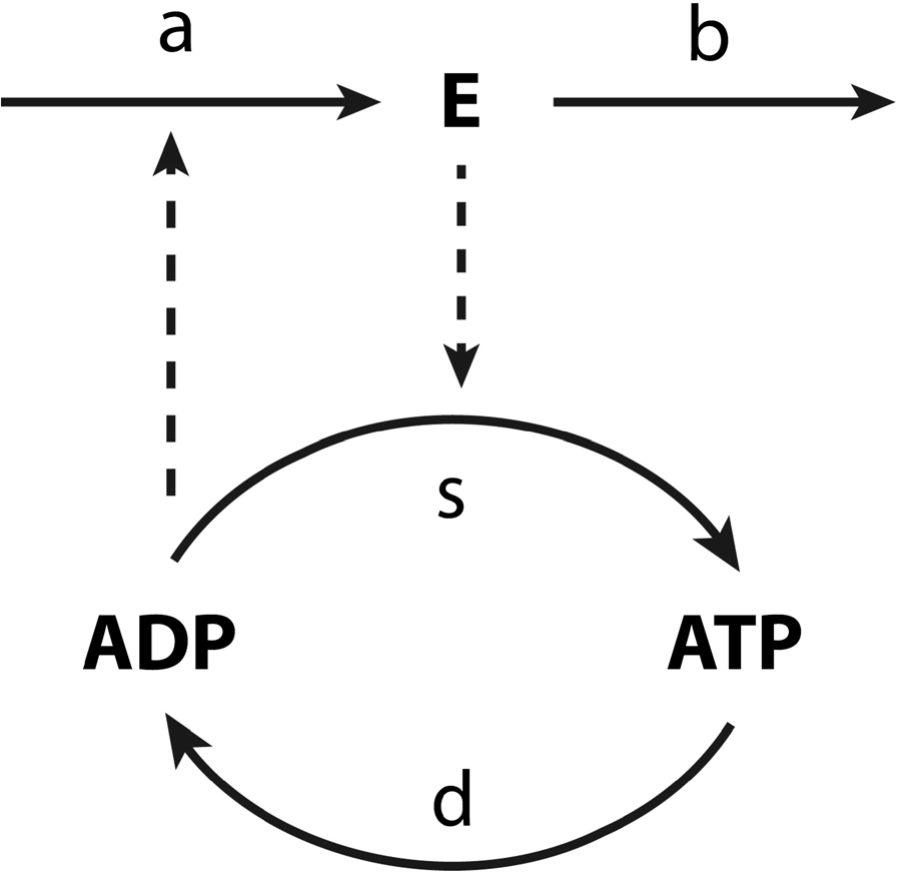
**ATP energy metabolism in a two-step pathway with gene-expression regulation.** The lower part represents a simplified ATP/ADP energy metabolism process, in which ADP acquires energy from the supply process (s) (i.e. phosphorylation) and produce ATP; also ATP can release energy in the demand process (d) (i.e. hydrolysis) and be converted to ADP. The supply is catalyzed by enzyme *E* (or enzymes stemming from an operon). The upper part describes the synthesis of enzyme *E* in process a and its degradation in process b.

Let us consider the simple example given in Figure 7, i.e. a two-step pathway with ATP and ADP as combined intermediate and with the expression of the gene encoding the first enzyme *E* increasing in proportion to the concentration of ADP. The ‘moiety conservation sum’ *C* is the sum of the concentrations of ATP and ADP and a constant here (i.e. *C* = [*ATP*] + [*ADP*]) because the reactions only convert the one into the other. The metabolic regulation addresses the interplay between the supply and demand processes (*s* and *d*).

The dynamics of ADP and enzyme *E* are here assumed to follow mass action kinetics:

(17) $$\begin{array}{l} d\left[\mathrm{ADP}\right]/\mathrm{dt}=-k_{s}\cdot E\cdot [\mathrm{ADP}]+k_{d}\cdot (C-[\mathrm{ADP}])\\ \mathrm{dE}/\mathrm{dt}=k_{a}\cdot \left[\mathrm{ADP}\right]-k_{b}\cdot E-k_{0} \end{array}$$

**Figure 8:**
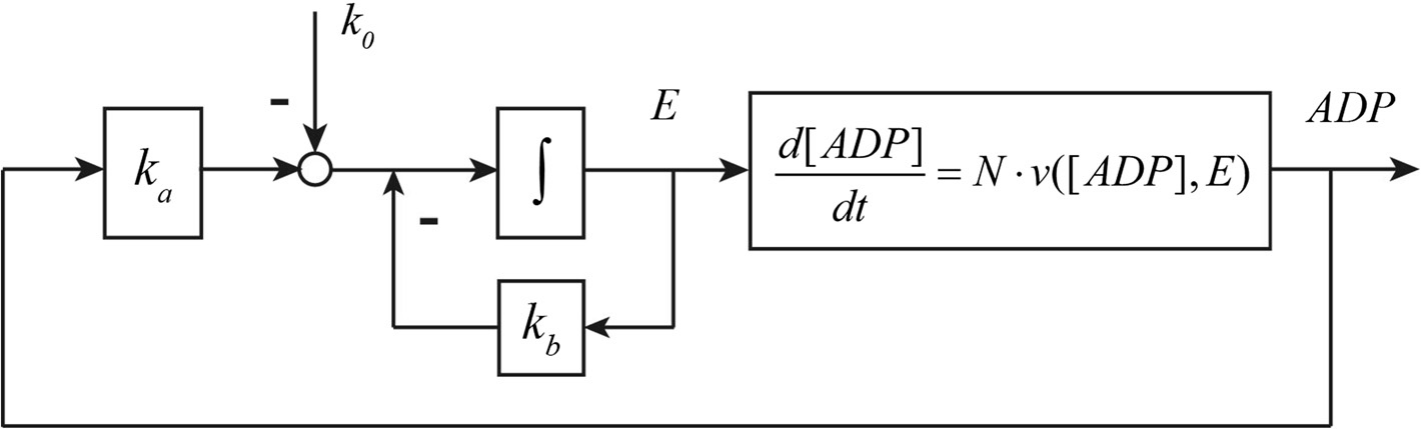
**Control system structure of ATP energy metabolism.***k*_0_ and *k*_b_ are the zero and first order protein degradation rate constants. *k*_a_ is the protein synthesis rate constant.

The degradation of the enzyme is here written as the sum of two terms, which will serve to emphasize the implication of this degradation to be independent (for *k*_*b*_ = 0) or dependent (for *k*_0_ = 0; see below) on the enzyme concentration. The first order degradation rate reflects the assumption that there is a rather unspecific protease activity for which the particular enzyme *E* we are considering here is a minority substrate. The zero order degradation would reflect a case where there is a specific protease system for enzyme *E* (e.g. an ubiquitination followed by a generic protease) that is saturated by the already high concentration of the enzyme relative to the *K*_*M*_ of the ubiquitin transferase. In (17) all rate constants are considered non-negative and *k*_0_ = 0 whenever *E* is non-positive. The closed-loop control system structure of the pathway can be represented as in Figure 8.

The control system diagram suggests that the ADP concentration is the controlled variable and the enzyme concentration *E* a manipulated variable in the gene-expression control loop. The zero order degradation rate *k*_0_ can be treated as a reference signal to the system. The metabolic regulation is included as a part of the ADP kinetic process. By considering a perturbation of *k*_*d*_ from its steady state value (i.e. *δk*_*d*_), and reformulating the kinetics of *ADP* and *E* (see Appendix C), we have

(18) $$\begin{array}{l} \delta \overset{\cdot }{\left[\mathrm{ADP}\right]}=-\left(k_{s}\cdot E_{\mathrm{ss}}+k_{d}\right)\cdot \delta [\mathrm{ADP}]-k_{s}\cdot [\mathrm{ADP}]{}_{\mathrm{ss}}\cdot \int_{0}^{\infty }\\ \;\left(k_{a}\cdot \delta \left[\mathrm{ADP}\right]-k_{b}\cdot \mathrm{\delta E}\right)\cdot \mathrm{dt}\\ \;+(C-{\left[\mathrm{ADP}\right]}_{\mathrm{ss}})\cdot \delta k_{d} \end{array}$$

Comparing this to a general closed-loop control system (16), with ADP for *X*, we recognize on the right-hand side first a proportional response term, then an integral response term, and then the perturbation term. The proportional response corresponds to the direct ‘elasticity’ of the supply and demand reactions with respect to the error function *δ*[ADP], which is a metabolic and instantaneous regulation. The integral response is related to the protein synthesis and degradation and thus to gene-expression regulation.

When the degradation of enzyme is zero order in terms of *E* (i.e. when *k*_*b*_ = 0), the gene-expression regulation becomes an ideal integral control loop, and the metabolic network can exhibit robust perfect adaptation to the external or parametric perturbations. This can be understood by requiring (18) to be valid in a steady state, i.e. with time independent values for [ADP] and *E.* Because the time integral reaches to infinity this requires that the argument of the integral, i.e. *k*_*a*_ ⋅ *δ*[*ADP*] - *k*_*b*_ ⋅ *δE*, must equal zero. The mechanism for this perfect adaptation is that after the perturbation the concentration of the enzyme will vary until it makes the time dependences equal zero by itself, forgoing the more usual process in metabolic regulation that changes in the controlled variable arrange for the steady state to be re-attained in the presence of the sustained perturbation.

For the more general case where the enzyme degradation may depend on the enzyme’s concentration, the (hierarchical) control of the enzyme level by the demand reaction can be expressed in terms of the kinetic parameters and the steady-state ADP concentration (see Appendix C):

(19) $$\begin{array}{l} H_{k_{d}}^{E}=\frac{\partial \ln E}{\partial \ln k_{d}}=\frac{\frac{k_{s}\cdot k_{a}}{k_{b}}\cdot {\left({\left[\mathrm{ADP}\right]}_{\mathrm{ss}}\right)}^{2}}{k_{d}\cdot C+k_{s}\cdot {\left({\left[\mathrm{ADP}\right]}_{\mathrm{ss}}\right)}^{2}\cdot \frac{k_{a}}{k_{b}}}\\ \;=1-\frac{1}{1+\frac{k_{s}\cdot {\left({\left[\mathrm{ADP}\right]}_{\mathrm{ss}}\right)}^{2}}{k_{d}\cdot C}\cdot \frac{k_{a}}{k_{b}}} \end{array}$$

The flux control exercised by the demand reaction is quantified by:

(20) $$H_{k_{d}}^{J}=\frac{\partial \ln J}{\partial \ln k_{d}}=1-\frac{\frac{{\left[\mathrm{ADP}\right]}_{\mathrm{ss}}}{C}}{1+\frac{k_{s}\cdot {\left({\left[\mathrm{ADP}\right]}_{\mathrm{ss}}\right)}^{2}}{k_{d}\cdot C}\cdot \frac{k_{a}}{k_{b}}}$$

Both the control of enzyme level and the control of demand flux by the perturbation equal 1 minus a hyperbolic function of *k*_*b*_. For the ideal integral control scenario of *k*_*b*_ = 0, the enzyme concentration *E* tracks the activity of the pathway degrading ATP perfectly, i.e. $H_{k_{d}}^{E}=1$. More importantly, the pathway flux perfectly tracks the perturbation in the demand flux and $H_{k_{d}}^{J}=1$. This is the case of robust perfect adaptation. For other cases when *k*_*b*_ ≠ 0, the adaptation of the pathway to the perturbation will not be perfect and both control coefficients are smaller than 1.

Also the robustness coefficient [36] of the ADP concentrations *vis-à-vis* perturbations in the demand reaction can be expressed in terms of kinetic constants and the concentration of ADP (see Table 1 for definition):

(21) $${ℜ}_{k_{d}}^{\left[\mathrm{ADP}\right]}=\frac{1}{\frac{\partial \ln\left[\mathrm{ADP}\right]}{\partial \ln k_{d}}}=\frac{k_{d}\cdot C+k_{s}\cdot {\left({\left[\mathrm{ADP}\right]}_{\mathrm{ss}}\right)}^{2}\cdot \frac{k_{a}}{k_{b}}}{\left(C-{\left[\mathrm{ADP}\right]}_{\mathrm{ss}}\right)\cdot k_{d}}$$

Only if *k*_*b*_ = 0, *k*_*d*_ = 0, or [ATP]_*ss*_ =0, the ADP and ATP are perfectly robust ( ${ℜ}_{k_{d}}^{\left[\mathrm{ADP}\right]}=\infty$) versus perturbations. The fragility [36] of the ADP concentration *vis-à-vis* perturbation in the demand reaction, which is the inverse of the robustness, can be quantified by the concentration control coefficient for the concentration of ADP with respect to the degradation process. It reads as:

(22) $$F_{k_{d}}^{\left[\mathrm{ADP}\right]}\equiv 1/{ℜ}_{k_{d}}^{\left[\mathrm{ADP}\right]}=\frac{\partial \ln\left[\mathrm{ADP}\right]}{\partial \ln k_{d}}=\frac{C-{\left[\mathrm{ADP}\right]}_{\mathrm{ss}}}{C+{\left({\left[\mathrm{ADP}\right]}_{\mathrm{ss}}\right)}^{2}\cdot \frac{k_{s}\cdot k_{a}}{k_{d}\cdot k_{b}}}$$

**Figure 9:**
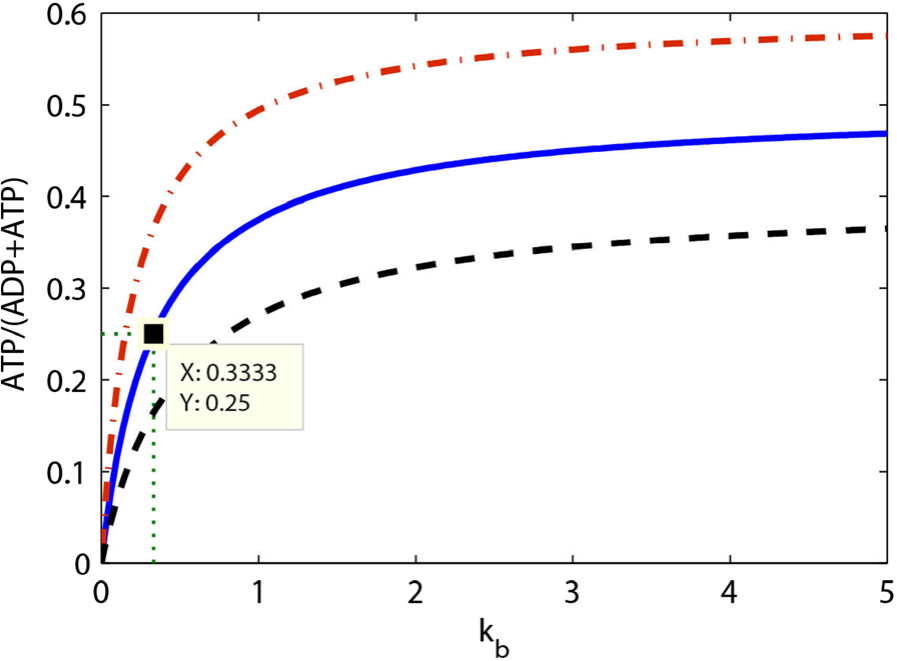
**The fragility of the ADP concentration with respect to perturbation in the demand flux as the protein degradation rate constant *k***_***b ***_**changes.** The fragility is zero when the *k*_*b*_ is zero; the maximum fragility equals the ATP/(ADP + ATP) ratio. The solid line represents the fragility under a ATP/(ADP + ATP) ratio of 0.5 at steady state; the dot-dash line is with a ratio of 0.4; and the dashed line is with a ratio of 0.6. The half maximum fragility is attained when *k*_*b*_ satisfies (23), e.g. *k*_*b*_ = 0.3333 under a ATP/(ADP + ATP) ratio of 0.5.

This fragility is a hyperbolic function of the first order degradation rate constant of the enzyme and hence zero when that degradation is zero-order (see Figure 9 for illustration). The fragility has the ATP/(ADP + ATP) ratio as its maximum value (hence the minimum robustness equals the (ADP + ATP)/ATP ratio). Half maximum fragility is attained for:

(23) $$k_{b}=\frac{{\left({\left[\mathrm{ADP}\right]}_{\mathrm{ss}}\right)}^{2}}{C}\cdot \frac{k_{s}\cdot k_{a}}{k_{d}}$$

This means that the fragility may be low for a substantial magnitude of the first order rate constant of protein degradation if the rate constant for protein synthesis is also high. The control coefficients for the enzyme level $H_{k_{d}}^{E}$ and the demand flux $H_{k_{d}}^{J}$ attain a maximum of 1 and a value of ½ when the fragility of ADP is half maximal.

These conclusions can be generalized somewhat by directly implementing HCA and hierarchical supply–demand result given in (12). For the above model the elasticity coefficients assume the following magnitudes:

(24) $$\begin{array}{l} \epsilon_{\left[\mathrm{ADP}\right]}^{s}=\epsilon_{\left[\mathrm{ADP}\right]}^{a}=\epsilon_{E}^{s}=1\\ \epsilon_{E}^{a}=0\\ \epsilon_{\left[\mathrm{ADP}\right]}^{d}=-\frac{\left[\mathrm{ADP}\right]}{C-\left[\mathrm{ADP}\right]} \end{array}$$

For the robustness of the ADP concentration vis-à-vis increased demand one then finds:

(25) $$\frac{1}{H_{d}^{\left[\mathrm{ADP}\right]}}=-\epsilon_{\left[\mathrm{ADP}\right]}^{d}+\epsilon_{\left[\mathrm{ADP}\right]}^{s}+\frac{\epsilon_{E}^{s}\cdot \epsilon_{\left[\mathrm{ADP}\right]}^{a}}{\epsilon_{E}^{b}-\epsilon_{E}^{a}}=\frac{C}{C-\left[\mathrm{ADP}\right]}+\frac{1}{\epsilon_{E}^{b}}$$

This shows that robustness is infinite if the enzyme degradation reaction is zero order (i.e. $\epsilon_{E}^{b}=0$), and that the robustness becomes smaller with increasing order (elasticity) of this reaction.

The most important conclusion here is that there is no discontinuity in the ability of integral control loops to lead to good adaptation. The closer the degradation of the enzyme that enables the adaptation is to a zero-order reaction, the stronger its tracking of the perturbation in the demand flux, and the higher the robustness of the variable that is to be kept homeostatic. Important perhaps is the phenomenon that the robustness is not determined by the magnitude of the degradation reaction but by its kinetic order (i.e. elasticity of effective Hill coefficient). The corresponding conclusions pertain to the tracking of the demand by the enzyme level *E* and the control of the pathway flux by the demand reaction.

A further issue in control engineering is the robustness of systems versus perturbations at various frequencies. In engineering, an airplane wing has to be robust to variations of air pressures at high frequencies, as well at low frequencies. In order to achieve this combined robustness, different control loops may have to be put in place simultaneously, although a trade-off limits what one can do [18]. In systems biology, this can be illustrated for the end-product feedback regulation in Figure 1. If the flux demand of the pathway increases rapidly, the concentration of the end product decreases rapidly and as a result of the direct allosteric product inhibition effect, the activity of the first enzyme will increase quickly too. This metabolic control of enzyme activity is a fast actuator of the system. However, if there is a further increase in the flux demand, the first enzyme may ‘lose’ its control capacity since its activity may be approaching its maximum capacity (*k*_cat_). At this stage, the system may then undergo a second ‘adaptation’ through gene expression which should be expected to be slower because the cell has to produce enzyme, but still ultimately lead to an increase in the concentration of the first enzyme. This increase should then decrease the direct metabolic stimulation of the catalytic activity of the enzyme discussed above. In this sense, the regulation of the first enzyme of the pathway is bi-functional in dynamic terms [18]: The metabolic regulation rapidly rejects high frequency perturbations but possibly with small amplitude or capability, while the gene-expression regulation is slow to adapt, but may be able to reject very large constant perturbations.

### Conditions for integral and pseudo-integral control: end-product pathway

**Figure 10:**
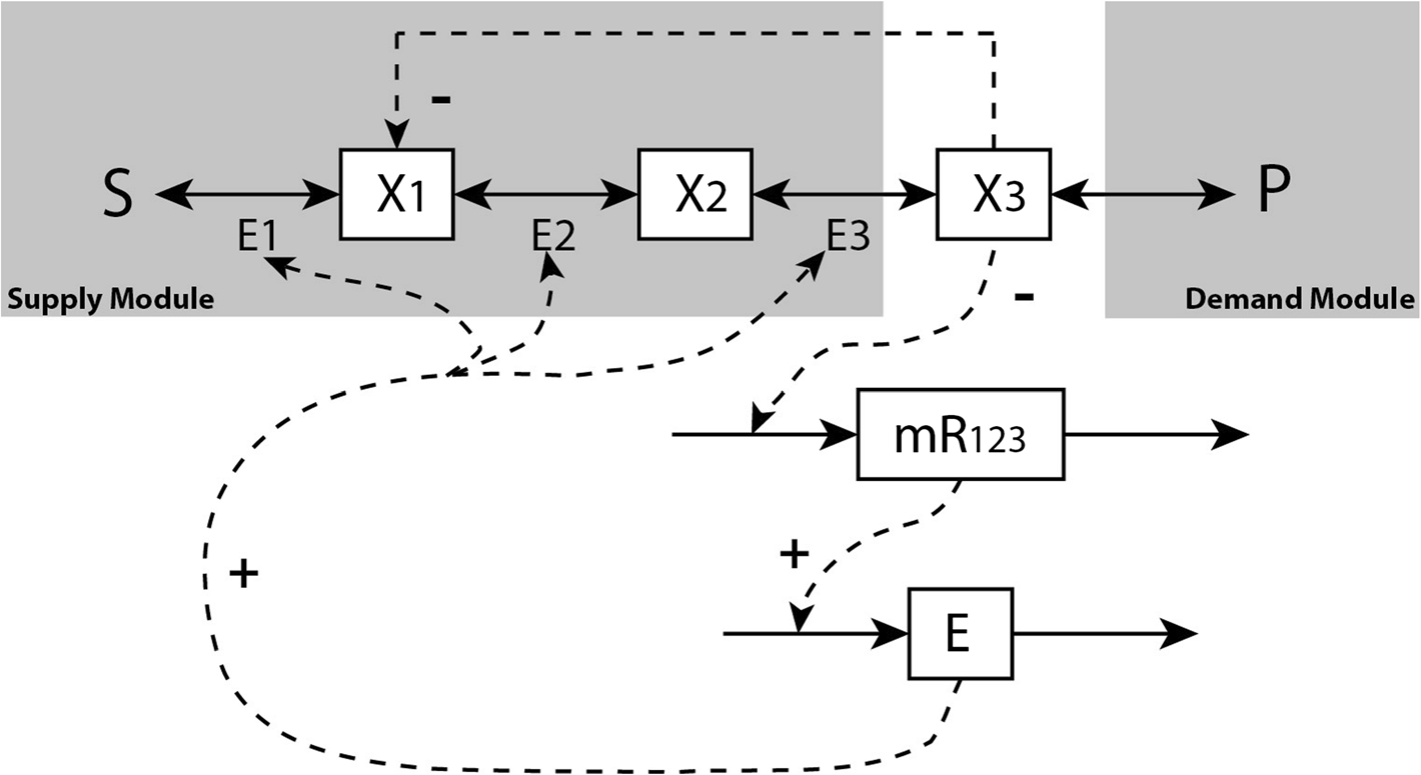
**A linear end-product pathway with gene-expression and metabolic regulation.** The metabolites are denoted by *x*_*i*_, mRNA by mR and enzymes by E. S and P are the external metabolites. Metabolite *x*_3_ inhibits the rate of enzyme 1 through metabolic regulation and the synthesis of enzyme 1, 2 and 3 through gene-expression regulation. Enzymes 1, 2 and 3 are encoded on the same operon.

In this section, we recall the end-product module example (of which the simple ATP metabolism example is a special case) to analyse gene-expression regulation in a more general metabolic pathway. In particular, the conditions will be identified for which the gene-expression regulation constitutes ideal integral control or pseudo-integral control will be discussed. A simple but representative end-product example is given in Figure 10, i.e. a linear pathway with three metabolites, where for all the three enzymes the gene expression is regulated by the last metabolite. The kinetic model of this example with all the parameters is provided in [5].

Gene transcription and translation are modelled here explicitly by further including the dynamic function of mRNA as

(26) $$\begin{array}{l} \dot{R}=v_{\text{Trsc}}-v_{\mathrm{RD}}=g_{\text{Trsc}}\left(x_{3}\right)-k_{\mathrm{RD}}\cdot R\\ \dot{E}=v_{\text{Trnl}}-v_{\mathrm{ED}}=g_{\text{Trnl}}\left(R\right)-k_{\mathrm{ED}}\cdot E \end{array}$$

Here *g*_Trnl_(*R*) = *k*_Trnl_ ⋅ *R* is a function of mRNA concentration *R. k*_ED_ essentially consists of three parts. One term is due to dilution, which is proportional to the specific cell growth rate *μ*. The other terms correspond to proteolysis, as below:

(27) $$k_{\mathrm{ED}}=\mu +k_{\text{proteolysis}}^{1}+k_{\text{proteolysis}}^{0}/E$$

The final term denotes the zero order proteolysis as would be caused by proteases that are saturated with the protein of interest. We here assume that the specific growth rate *μ* is independent of the activity of the pathway under study, an assumption that is sometimes but not always realistic. Since after multiplying with *E* the last term is independent of the protein concentration, it is convenient to move this term into the protein synthesis function *g*_Trnl_(*R*). Hence, the new protein degradation rate can be defined as,

(28) $$k_{\mathrm{ED}}=\mu +k_{\text{proteolysis}}^{1}$$

and the protein dynamics can be re-written as,

(29) $$\begin{array}{l} \dot{E}=g_{\mathrm{Trnl}}\left(R\right)-k_{\mathrm{ED}}\cdot E\\ =\left(k_{\mathrm{Trnl}}\cdot R-k_{\text{proteolysis}}^{0}\right)-\left(\mu +k_{\text{proteolysis}}^{1}\right)\cdot E \end{array}$$

**Figure 11:**
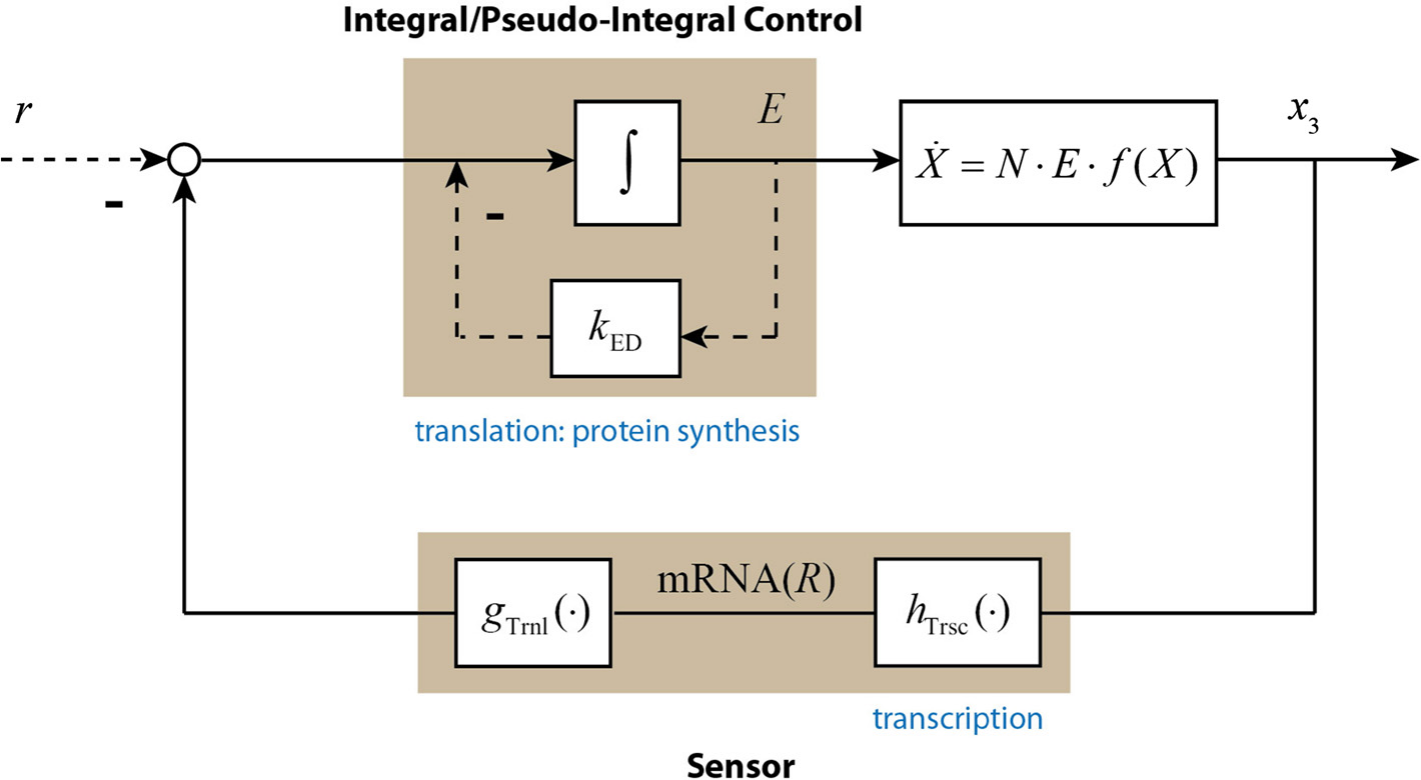
**Control system structure of a pseudo-integral or an ideal integral control problem. ***h*_Trsc_(·) denotes the transcription process. *g*_Trnl_(·) denotes the rate of protein synthesis. The pseudo-integral control system becomes an ideal integral control only when the dashed line connecting the degradation rate *k*_ED_ is removed.

In the exponential growth phase or if proteolysis is first order (i.e. *k*_ED_ ≠ 0), the above pathway example corresponds to a pseudo- or non-integral control scenario. The control structure of the regulatory system is then given by Figure 11. The dynamics of the ‘sensor’ is decomposed here by addressing both transcription and the translation through mRNA. At steady state, often *g*_Trnl_(*R*)_*ss*_ = *k*_ED_ ⋅ *E*_*ss*_ ≠ 0. Therefore, after perturbation of a system parameter (i.e. kinetic constants), the new steady state values of *x*_3_, *R*, and *g*_Trnl_(*R*) will no longer be the same as the old steady state values, which indicates that then the regulatory system does not achieve perfect adaptation. However, when *k*_TnD_ is very small, near-perfect adaptation behaviour should be observed.

An ideal integral control scenario will happen only when cell enters a stationary phase and there only exists zero order proteolysis, i.e. $k_{\mathrm{ED}}=\mu +k_{\text{proteolysis}}^{1}=0$. In such case, at steady state, *g*_Trnl_(*R*)_*ss*_ ≡ 0 due to integral control, and *R* and *x*_3_ will also always keep the same constant values at steady state, no matter how the system parameters are perturbed. The adaptation of the system is then perfect in the sense of making the system properties *R* and *x*_3_ robust against the perturbations. The functions *g*_Trnl_(·) and *g*_Trsc_(·) can also be multivariate, i.e. contain other system variables. Only if those variables do not depend functionally on the protein concentration (*E*), the adaptation *vis-à-vis* perturbations of the system kinetic parameters will remain perfect.

The ideal integral control scenario that we considered above led to the perfect robustness of certain system variables with respect to certain (external or parametric) perturbations. A second aspect of the perfect adaptation scenario is the perfect tracking by a second system variable of perturbations. Perfect tracking means that the relative change in the variable is identical to the relative change in the perturbing parameter. If the parameter is the activity of a process, then this means that the corresponding control coefficient is equal to 1. The perfect tracking of references and the perfect robustness of controlled variables to perturbations are two aspects of integral control systems, i.e. the two features can be observed simultaneously for the same system. A specific pathway then shows perfect robustness of a system variable *vis-à-vis* multiple perturbations (or parameters) but perfect tracking with respect to only a limited set of parameters (e.g. a reference signal *r*).

For the aforementioned example, the zero order proteolysis can be regarded as a ‘reference’ signal $r=k_{\text{proteolysis}}^{0}$. Effectively perturbation of this rate constant corresponds to an external perturbation of protein synthesis. The effect is that even at an ideal integral control case, i.e. when *k*_ED_ = 0, the steady state concentration of mRNA will not become 0. Rather, it will always ‘track’ the reference *r* (i.e. *R* = *r*/*k*_Trnl_ ≠ 0) whenever ${\dot{E}}_{\mathrm{ss}}=0$. The reference tracking control system structure is included in Figure 11 with reference *r* referred to by a dashed arrow, and with *g*_Trnl_(*R*) then representing *k*_Trnl_ ⋅ *R*.

#### Practical concerns and assumptions on degradation rate constant k_ED_

In general, $k_{\mathrm{ED}}=\mu +k_{\text{proteolysis}}^{1}>0$. It may seem that the condition of integral control can be approached, when either i) *k*_ED_ < <*α* with *α* a very small positive value, or ii) *k*_ED_ < <*k*_Trnl_ (or *k*_ED_ *· E* < <*g*_Trnl_(*R*)). In the latter case, for the level of protein to remain bounded, there should be a background degradation rate of the protein independent of the concentration of that protein. This would be so if:

(30) $$\mu +k_{\text{proteolysis}}^{1}\ll k_{\text{proteolysis}}^{0}/E$$

During the exponential growth phase of bacteria such as *E. coli* less than 1% of a protein may be degraded during a cell division cycle [37]. Consequently the major term in the protein degradation is the dilution term *μ* and this term is generally very small. Indeed, by definition, *e*^*μ* ⋅ *T*^ = 2, where *T* is the doubling time of the bacteria and then *μ* = ln2/*T*. Practically, the smallest value for *T* is close to 20 minutes [38], which corresponding to the fastest growth rate of *E. coli*:

(31) $$\mu \approx \ln2/\left(20\times 60\right)=5.8\times 10^{-4}\;\left(s^{-1}\right)$$

Such a growth rate in microbes such as *E. coli* and yeast, which is fast for organisms but slow at the time scale of RNA and protein synthesis, produces a small effective degradation rate constant for the proteins. Below we shall see whether such a small degradation rate constant suffices to produce near-perfect adaptation behaviour in practice, which would be interpreted as a quasi-perfect integral control system.

#### Simulation study

**Figure 12:**
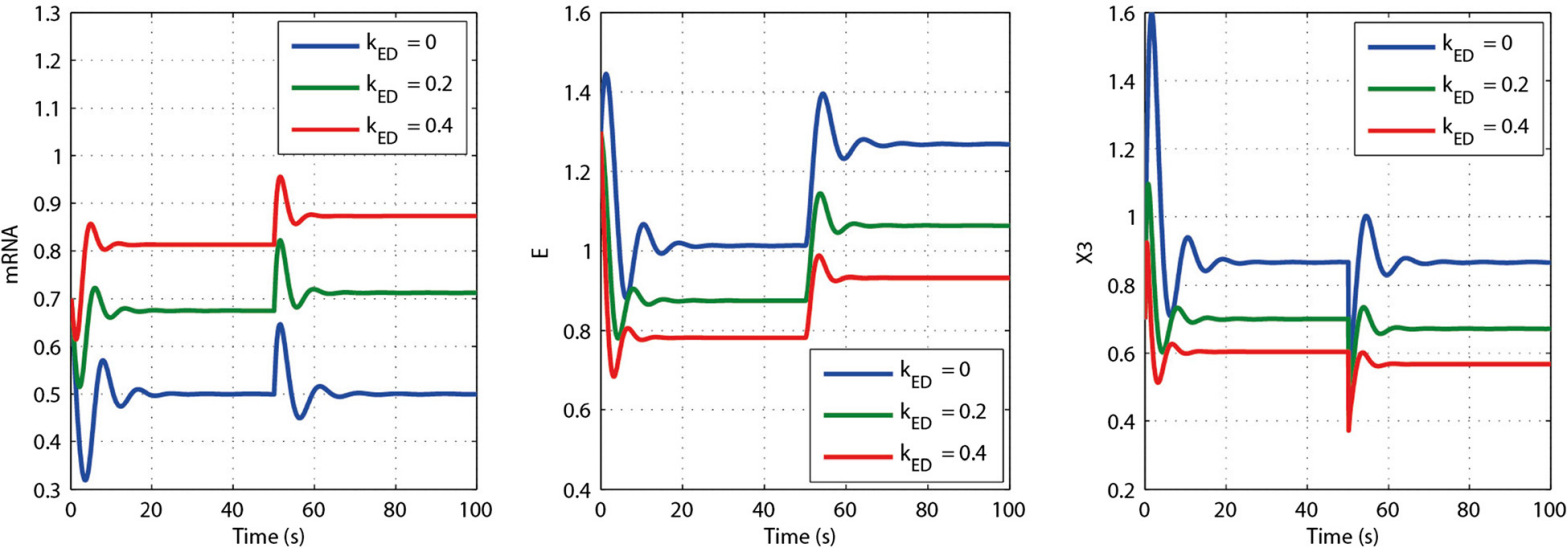
**The responses of mRNA, enzyme (*E*), *x***_**3 **_**under perturbation with different *k***_**ED **_**values.**

In this section, both the integral and the non-integral control scenarios are simulated based on the example given in Figure 10 with all the kinetic parameters given in [5]. Three different systems are considered, with *k*_ED_ = 0, 0.2, and 0.4, respectively. All three systems are simulated from the same initial condition. The concentration changes of mRNA, *E* and *x*_3_ are shown in Figure 12. After a period of time (e.g. 30 seconds) the three systems reach different steady states. Then at 50 seconds we perturb one system parameter (i.e. the *k*_cat_ of the third reaction) by 20% for all three cases. After a while the three systems reach new steady states. After perturbation only the system with zero order protein degradation (i.e. *k*_ED_ = 0), returns to the same steady state values for mRNA and *x*_3_ as those before perturbation, which indicates perfect adaptation: this is an ideal integral control system. In this case the manipulated variable, the enzyme level *E*, varies strongly. Its adaptation enables *x*_3_ and mRNA to be completely robust vis-à-vis the perturbations of the *k*_cat_ of the third reaction. In the other two cases (i.e. *k*_ED_ = 0.2 or 0.4), the change in enzyme level is smaller, but the new steady state values of mRNA and *x*_3_ deviate from their previous steady-state values and adaptation is not perfect. However, the deviations are not large; only 3% for *x*_3_ and 4% for mRNA when *k*_ED_ = 0.2, and 4% for *x*_3_ and 8% for mRNA when *k*_ED_ = 0.4.

**Figure 13:**
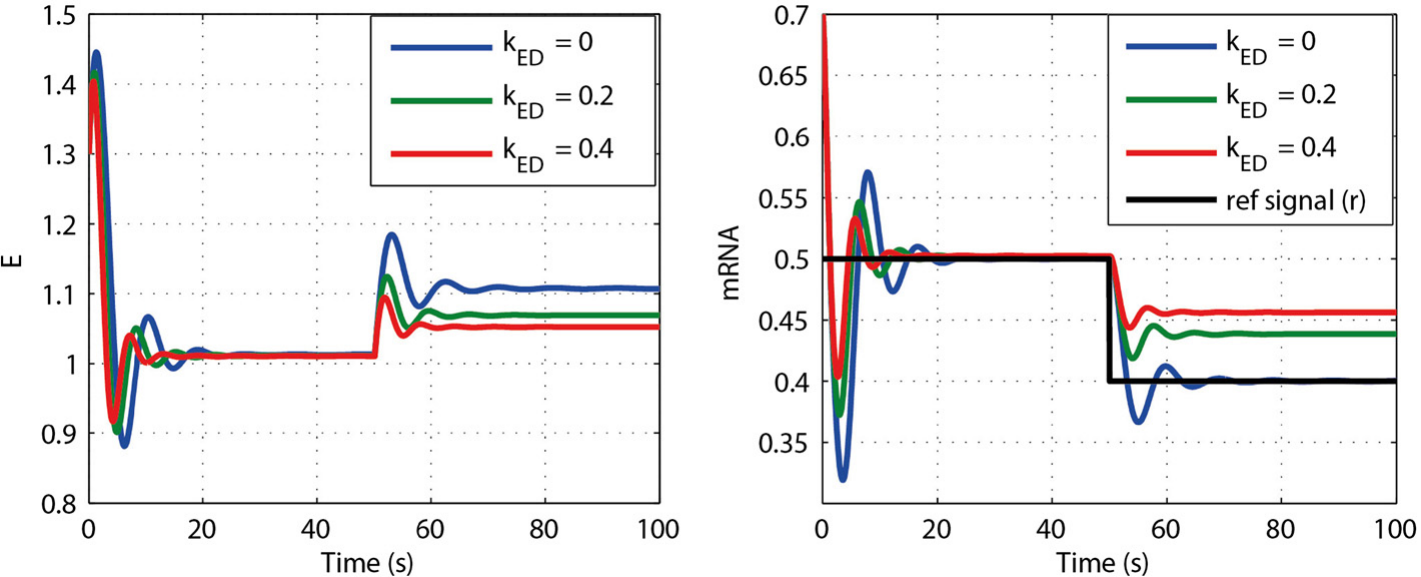
**The responses of enzyme (*E*) and mRNA concentrations with different *k***_**ED **_**values.** First the reference signal *r* was set to 0.5. By adjusting the protein synthesis rate (*k*_trnl_), the same steady state values of mRNA (i.e. mRNA_ss_ = (*k*_ED_ · *E*_ss_ + *r*)/*k*_trnl_ = 0.5) and enzyme concentrations were produced. At *t* = 50 seconds, the reference signal *r* was changed from 0.5 to 0.4.

Now let us consider the reference tracking scenario. The responses of enzyme and mRNA to a 20% perturbation in the protein stability (*r*) at *t* = 50 seconds, are given in Figure 13 for three different rate constants of enzyme degradation *k*_ED_. Only when *k*_ED_ = 0 the mRNA concentration tracked the reference value with zero steady state ‘deviation’. This indicates the existence of a perfect integral action of the feedback regulatory system. When *k*_ED_ was not equal to zero (i.e. *k*_ED_ = 0.2 or *k*_ED_ = 0.4), the mRNA response did not track the reference signal, indicating that in these two cases the controller of the system was not an ideal integral controller, although it changed less than did the reference signal.

#### HCA and an hierarchical supply–demand interpretation

This simulation example can be represented by a hierarchical supply–demand structure such as in Figure 4. To obtain ideal integral control, both protein synthesis and protein degradation should be independent of the concentration of the protein that is being degraded (i.e. $\dot{E}=g_{\mathrm{Trnl}}\left(R\right)$). Since this implies that protein degradation is zero order in protein concentration:

(32) $$\epsilon_{E_{s}}^{b}=\epsilon_{E_{s}}^{a}=0$$

So that the hierarchical control coefficient of the metabolite concentration becomes

(33) $$H_{s}^{X}=\frac{1}{\epsilon_{X}^{d}-\epsilon_{X}^{s}-\frac{\epsilon_{E_{s}}^{s}\cdot \epsilon_{x}^{a}}{\epsilon_{E_{s}}^{b}-\epsilon_{E_{s}}^{a}}}=\frac{1}{\epsilon_{X}^{d}+\left(-\epsilon_{X}^{s}\right)+\frac{\epsilon_{E_{s}}^{s}\cdot \left(-\epsilon_{x}^{a}\right)}{0}}=0$$

Here $\epsilon_{E_{s}}^{a}=\epsilon_{E}^{v_{\mathrm{Trnl}}}$, $\epsilon_{E_{s}}^{b}=\epsilon_{E}^{v_{\mathrm{ED}}}$, $H_{s}^{X}=H_{3}^{x_{3}}$ for the biosynthetic pathway example. Because the hierarchical control by supply and demand must add to zero (due to the concentration control summation law [15]), also the control by demand on the metabolite concentration becomes precisely equal to zero in the zero order protein degradation case.

### Feed-forward activation: a case study of a leucine biosynthetic pathway

**Figure 14:**
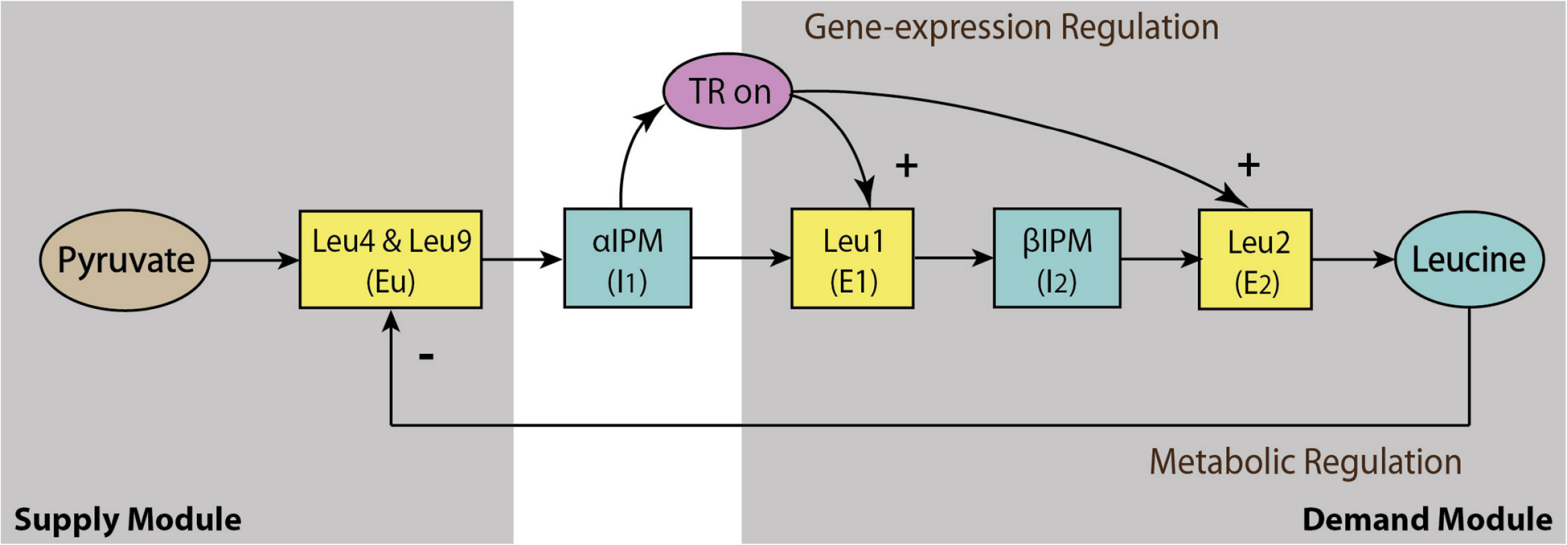
**A schematic diagram of the simplified leucine biosynthetic pathway model. ***E*_u_ represents Leu4 and Leu9, *E*_1_ represents Leu1, *E*_2_ represents Leu2, *I*_1_ and *I*_2_ denote αIPM (α-isopropylmalate) and βIPM respectively. The source is pyruvate and *P* represents leucine. The supply and demand modules of the pathway are shown in shading.

In previous sections, either a metabolic intermediate (ADP) or a penultimate product (*x*_*n*_) inhibited or repressed upstream enzymes. In this section a different regulatory structure is investigated, one in which a metabolite activates downstream enzymes through gene-expression. A simplified mathematical model describing the leucine biosynthetic pathway in *Saccharomyces cerevisiae*[39] is used to demonstrate that the analysis integrating hierarchical supply–demand theory and control engineering continues to apply. The pathway converts pyruvate to leucine by the sequential reactions described in Figure 14. There are two major regulatory mechanisms in the pathway. One is a metabolic feedback inhibition of Leu4 and Leu9 by leucine, which is an end-product module similar to the ones discussed above. The other is the transcriptional (gene-expression) activation of downstream enzymes Leu1 (*E*_1_) and Leu2 (*E*_2_) by αIPM (*I*_1_) (through transcription factor Leu3), which we shall call initial-product modules (see Appendix A and Figure 17). The model predictions fit the experimental data and all the parameter values have been estimated and provided in [39].

**Figure 15:**
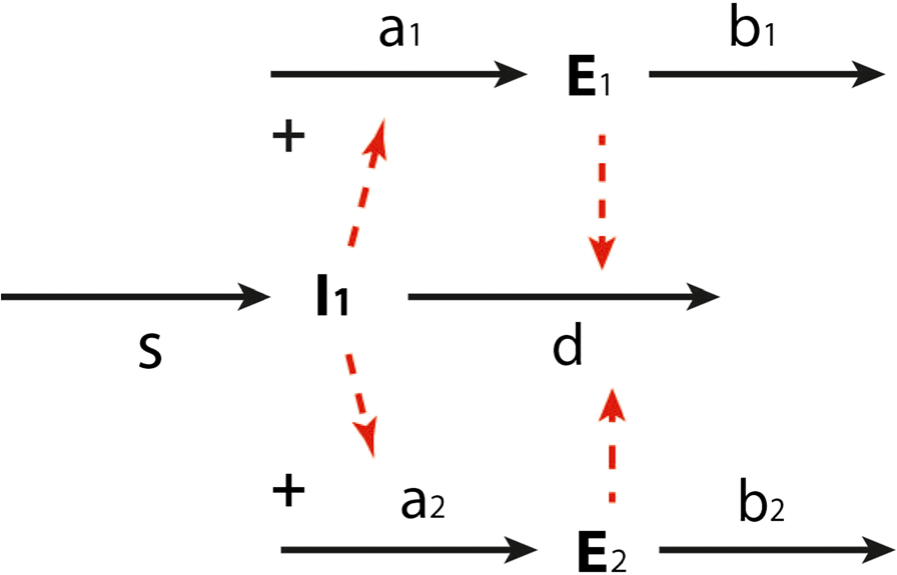
**Illustration of the hierarchical supply–demand structure of the leucine biosynthetic pathway.** a_1_ and b_1_ denote the synthesis and degradation of enzyme Leu1 (*E*_1_); a_2_ and b_2_ denote the synthesis and degradation of enzyme Leu2 (*E*_2_). s and d represent the supply and demand modules as shown in Figure 14.

The hierarchical control coefficient quantifying the control of supply enzymes *E*_s_ with respect to the concentration of *I*_1_ (αIPM) can be expressed into the various elasticity coefficients (see also Figure 15),

$$\begin{array}{l} H_{s}^{I_{1}}=\frac{1}{\left(\epsilon_{I_{1}}^{d}+\epsilon_{E_{1}}^{d}c_{a_{1}}^{E_{1}}\epsilon_{I_{1}}^{a_{1}}+\epsilon_{E_{2}}^{d}c_{a_{2}}^{E_{2}}\epsilon_{I_{1}}^{a_{2}}\right)-\epsilon_{I_{1}}^{s}}\\ \;=\frac{1}{\epsilon_{I_{1}}^{d}-\epsilon_{I_{1}}^{s}+\frac{\epsilon_{E_{1}}^{d}\cdot \epsilon_{I_{1}}^{a_{1}}}{\epsilon_{E_{1}}^{b_{1}}-\epsilon_{E_{1}}^{a_{1}}}+\frac{\epsilon_{E_{2}}^{d}\cdot \epsilon_{I_{1}}^{a_{2}}}{\epsilon_{E_{2}}^{b_{2}}-\epsilon_{E_{2}}^{a_{2}}}} \end{array}$$

and its value can be computed from the simulation of the pathway model by perturbing *E*_*u*_ and recording the changes in the steady state value of *I*_1_. This led to $H_{s}^{I_{1}}=0.12$. The hierarchical control coefficient is small but not zero, which indicates that the regulatory network can react and adapt to this external perturbation although the adaptation is not ‘perfect’.

The closed-loop control system structure of the downstream gene-expression activation is the same as that of the upstream inhibition case described in Figure 11 (with end-product metabolic regulation dominating process dynamics). It is still a negative feedback control rather than a feed-forward control in the control context: For a *negative* feedback control system, a change (increase/decrease) in some controlled variable will result an opposite change (decrease/increase) in the operation of the process itself in such a way as to reduce changes. For the end-product module given in (1), when *E*_1_ increases the concentration of the end product *x*_*n*_ also increases, whilst as *x*_n_ increases *E*_1_ would decreases as a result of negative feedback inhibition (through metabolic or gene-expression regulation). Hence this is a negative feedback control system, since when *E*_1_ increases the system attempts to reduce such an increase. For case of the positive activation of downstream enzyme by a metabolite upstream of that enzyme (i.e. initial-product module given in (34)), when *E*_1_ increases the concentration of *x*_1_ decreases since it is a downstream enzyme, while *E*_1_ would also decreases as *x*_1_ decreasing because of the positive feed-forward activation. This gives the same result as the end-product case. Herewith, both two systems are negative feedback control system in the control context and both can be represented by the same feedback control structure as given in Figure 11.

**Figure 16:**
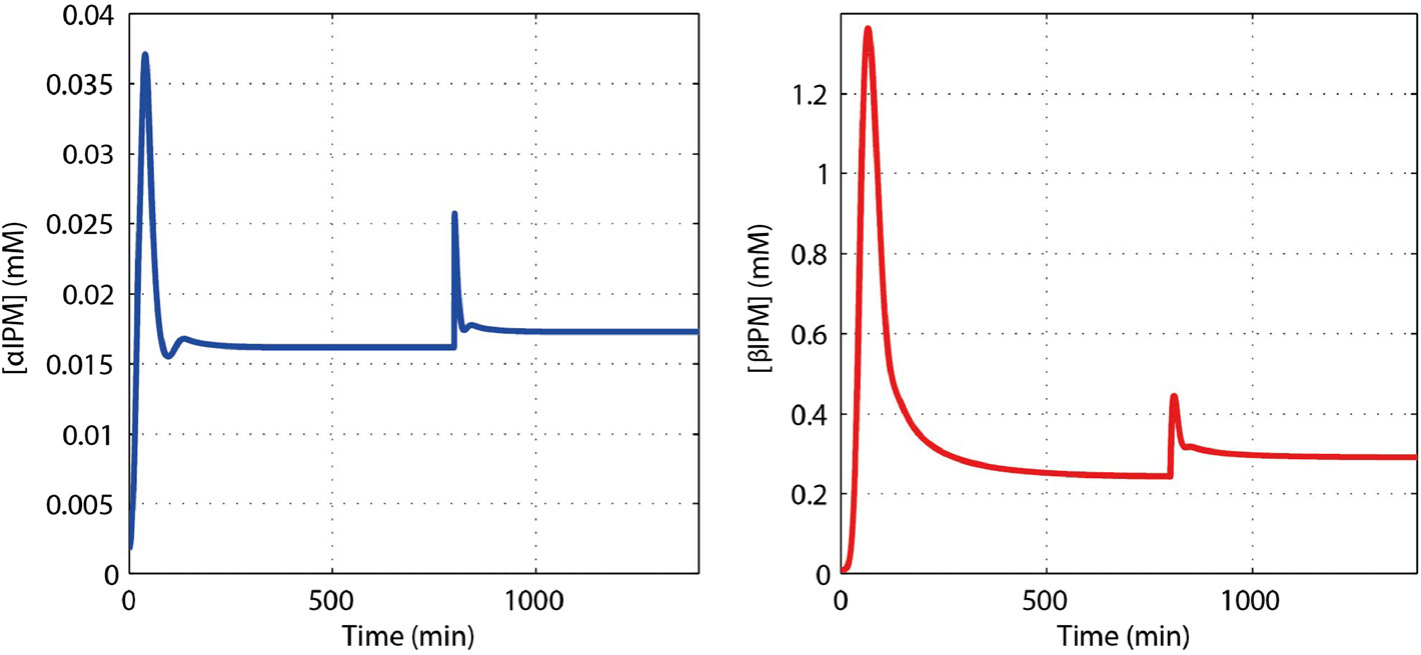
**Concentration changes of αIPM (*I***_**1**_**) and βIPM (*I***_**2**_**) under perturbation (at 800 min).**

In this model, the protein degradation rates purely depend on the dilution effects, and the estimated growth rate of *μ* = 0.0058 min^-1^ = 9.7 × 10^-5^ s^-1^ is very small as compared to metabolic turnover times and may imply the existence of a quasi-integral control scenario. For testing, a simulation study is provided where a 50% increase on the *k*_cat_ of the first reaction is applied at 800 minutes as an environmental perturbation, the calculated concentration changes of the intermediate metabolites are shown in Figure 16. This perturbation could be due to an allosteric activation by a substance added to the system. The concentrations of both αIPM and βIPM reach new steady states. The differences between the new and the old steady-state values are around 7% and 20% respectively. Again, the adaptation is imperfect; the two concentrations are robust but not infinitely so.

## Conclusions

In this paper, two existing approaches to the analysis of the robustness and adaptation of networks have been integrated: control engineering and MCA. The former designs control structures for networks that lead to optimal behaviour, for instance in terms of ‘perfect adaptation’ leading to infinite robustness. The latter quantifies the extents to which processes in a network determine fluxes and concentrations and identifies the molecular interactions that determine the corresponding distributions of control. Two extensions of MCA have also been integrated into our analysis, i.e. HCA adding the possibility to analyse regulation through gene expression, and supply–demand theory, greatly simplifying the analysis towards understanding of the essence. We also integrated the two latter approaches into a novel hierarchical supply–demand theory. The steady state properties of exemplary metabolic pathways served as test cases; they were analysed in terms of the robustness of their steady state properties. They included a pathway of free energy transduction, a pathway with feedback inhibition and repression, and a pathway with feed-forward regulation. Most substrates for the synthesis of macromolecules such as proteins and nucleic acids are the end product of such pathways, making this analysis important for the understanding for the control of cell growth.

We then used the resulting framework to address the question whether metabolic pathways regulated by both metabolic interactions and through gene expression, come close to the ideal control structures designed in control engineering. We focused on the control structures leading to so-called ‘perfect adaptation’ , as defined by (i) complete robustness of the concentration of the pathway’s end product towards perturbations in supply and demand, (ii) perfect tracking of perturbations by variables involved in the adaptation. Control engineering distinguishes between proportional, derivative and integral control and showed that of these only integral control loops produce perfect adaptation. In a hierarchical control analysis, in a hierarchical supply–demand analysis, as well as in a number of computer simulations, we showed that such perfect adaptation (and perfect tracking) should not be expected for the usual gene-expression regulation pathways, even though they seem to engage in integral control. Although that integral control increased the robustness of the concentration of the pathway product vis-à-vis perturbation in the activities of metabolic enzymes, that robustness was not perfect, except for the singular case where the metabolic enzymes would be infinitely stable. For complete steady states, such infinite stability should not be considered realistic.

We expected that for cases where proteins are highly, adaptation of the anabolic networks studied should be high even if not quite perfect. This was however not much observed. HCA can show that this is not to be expected either. The perfectness of the adaptation of the networks in complete steady state should not be a function of the magnitude of any first-order protein degradation rate constant, but rather a function of the order of the protein degradation reaction, or to be precise, of its elasticity coefficient: the protein degradation should be zero order in protein concentration for the adaptation to become perfect.

Because gene expression regulation involves a time integral that is a function of changes at the level of the metabolic pathway, we suggest that at least when applied to biological systems, control engineering is extended so as to explicitly include such ‘integral control’ loops even if they do not lead to perfect adaptation. By adding the subtlety of HCA one can then analyse how perfect the adaptation furnished by the integral control loop actually is. We propose a simplification of the control-engineering concept of ‘proportional control’ to the more general case where the adaptation is any function of the displacement of the controlled variable but neither a function of the time integral nor the time derivative of that displacement. That function could be more complex than the proportionality used in control engineering and thereby be realistic for biochemical networks, thereby making control engineering much more useful for the life sciences. Perhaps the terms ‘proportional control’ should then be replaced by ‘direct or metabolic control’ , referring to the direct nature of the interactions.

Metabolic networks typically have more than one metabolic intermediate and when the network is perturbed by affecting a process activity, many metabolite concentrations tend to change. If it is of particular interest to maintain one of these as constant as possible whereas variations in other metabolite concentrations are less detrimental to biological function, then the former may be designated as the ‘controlled variable’ and the latter as ‘manipulated variables’ in the control engineering analysis. Because many enzymes do not serve a function other than through the reaction they catalyse, they may be the more obvious ‘manipulated variables’. On the other hand it may well be that some metabolite concentrations serve as ‘manipulated variables’ with the sole function of providing near perfect control loops. cAMP might be an example.

Integral control loops do not require gene-expression regulation. Also in exclusively metabolic networks, integral control may arise. An example would be a linear pathway with the penultimate metabolite affecting the first reaction of the pathway, whilst its degradation rate is independent of its concentration. If the first step of the pathway is then perturbed, the concentration of the penultimate metabolite will be a function of the time integral of the perturbation of the first metabolite concentration in the pathway, and it may effect perfect adaptation. On the other hand, if the degradation rate of that penultimate metabolite were first order, then that concentration would be a direct function of the concentration of the first metabolite of the pathway, and the regulation would turn into proportional rather than integral control. In hindsight, reference [33] was an early example of this.

As shown at length in the present paper, gene-expression regulation does not always lead to perfect adaptation. Indeed, if protein degradation is first order, then the deviation in the concentration of the enzyme may well be proportional to the perturbation in the controlled variable and one effectively obtains ‘proportional control’ through gene-expression regulation. We conclude that whether control loops correspond to the integral or proportional category of control engineering depends on the elasticity coefficients (orders) of the reactions involved with respect to the controlled variable, rather than on time integration being involved. The extension of control engineering with hierarchical control analysis that was initiated here, may well provide the subtlety that helps analyse the complex networks that mankind is confronted with today, both in the life sciences and in economics and environmental sciences. It may also help design new and better networks, if only for synthetic biology and biotechnology.

We intend the present work to serve as a beginning of a development where multiple principles of control engineering may be compared with achievements by biological evolution. One example where the present work may be extended to is the case where rather than that one piece of DNA encodes three metabolic enzymes (as in Figure 8), one metabolic enzyme catalyses reactions in three metabolic modules in the metabolic network: a multifunctional enzyme. The gene expression of that enzyme may be regulated by metabolites in all three modules. This is a type of network structure that control engineering may come up with as serving a function of coordination. And it will be of interest whether this type of network may serve a similar function in Biology.

## Appendices

A. The initial-product module

**Figure 17:**
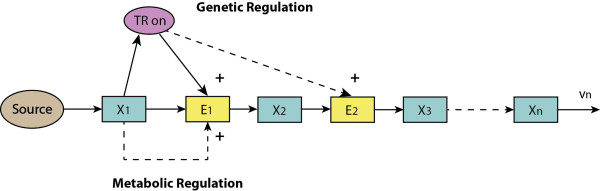
The initial-product module with gene-expression and metabolic regulation.

The initial-product feed-forward regulation module given in Figure 17 can be mathematically described by the following differential equations:

(34) $$\begin{array}{l} {\dot{x}}_{1}\left(t\right)=v_{0}(t)-E_{1}(t)f_{1}(x_{1}(t),p(t))\\ {\dot{x}}_{2}\left(t\right)=E_{1}(t)f_{1}(x_{1}(t),p(t))-E_{2}(t)f_{2}(x_{2}(t),x_{3}(t))\\ \vdots \;\vdots \;\vdots \\ {\dot{x}}_{n}\left(t\right)=E_{n-1}(t)f_{n-1}(x_{n-1}(t),x_{n}(t))-E_{n}(t)f_{n}(x_{n}(t))\\ {\dot{E}}_{1}\left(t\right)=g(x_{1}(t))-k_{\mathrm{ED}}E_{1}(t) \end{array}$$

The first reaction here is still assumed product insensitive and other factors *p* can act on the enzyme. Different from the end-product module, the function *g* is assumed to be an increasing function of its argument. It has been demonstrated in [28] that if a constant steady-state regimen exists, the following simple relationship should be satisfied, with ${\bar{E}}_{1}=g\left({\bar{x}}_{1}\right)/k_{\mathrm{ED}}$ and $\left(g\left({\bar{x}}_{1}\right)/k_{\mathrm{ED}}\right)\cdot f_{1}\left({\bar{x}}_{1}\right)=v_{1}$. Here, it is assumed the intermediate reactions of the metabolic pathways do not ‘saturate’.

## B. Calculation of global and local control coefficients in a supply–demand system

According to the summation and connectivity laws, for a *supply–demand* system, we have

$$\begin{array}{l} C_{d}^{J}+C_{s}^{J}=1\\ C_{d}^{x_{n}}+C_{s}^{x_{n}}=0\\ C_{d}^{J}\cdot \epsilon_{x_{n}}^{d}+C_{s}^{J}\cdot \epsilon_{x_{n}}^{s}=0\\ C_{d}^{x_{n}}\cdot \epsilon_{x_{n}}^{d}+C_{s}^{x_{n}}\cdot \epsilon_{x_{n}}^{s}=-1 \end{array}$$

By solving the above four equations, the ‘global’ concentration and flux control coefficients with respect to the *supply* and *demand* steps can be derived as

$$\begin{array}{l} C_{s}^{x_{n}}=\frac{1}{\epsilon_{x_{n}}^{d}-\epsilon_{x_{n}}^{s}},\;C_{d}^{x_{n}}=\frac{-1}{\epsilon_{x_{n}}^{d}-\epsilon_{x_{n}}^{s}}\\ C_{s}^{J}=\frac{-\epsilon_{x_{n}}^{d}}{\epsilon_{x_{n}}^{s}-\epsilon_{x_{n}}^{d}},\;C_{d}^{J}=\frac{\epsilon_{x_{n}}^{s}}{\epsilon_{x_{n}}^{s}-\epsilon_{x_{n}}^{d}} \end{array}$$

The expressions of the ‘local’ flux control coefficients given in (5) can be obtained by solving the following summation and connectivity laws with respect to the local linear pathway within the *supply* module as given in Figure 3.

$$\begin{array}{l} c_{1}^{J_{1}}+c_{2}^{J_{1}}+\cdots +c_{n-1}^{J_{1}}=1\\ c_{1}^{J_{1}}\epsilon_{x_{2}}^{v_{1}}+c_{2}^{J_{1}}\epsilon_{x_{2}}^{v_{2}}=0\\ \;\vdots \\ c_{n-2}^{J_{1}}\epsilon_{x_{n-1}}^{v_{n-2}}+c_{n-1}^{J_{1}}\epsilon_{x_{n-1}}^{v_{n-1}}=0 \end{array}$$

## C. Steady state analysis of the ATP metabolism example

The steady state values of ADP and *E* before the perturbation are determined when *d*[*ADP*]/*dt* = 0 and *dE*/*dt* = 0:

(35) $$\left\{\begin{array}{l} E_{\mathrm{ss}}=\frac{k_{d}\cdot \left(C-{\left[\mathrm{ADP}\right]}_{\mathrm{ss}}\right)}{k_{s}\cdot {\left[\mathrm{ADP}\right]}_{\mathrm{ss}}}\\ {\left[\mathrm{ADP}\right]}_{\mathrm{ss}}=\frac{k_{b}\cdot E_{\mathrm{ss}}+k_{0}}{k_{a}} \end{array}\right)$$

and

(36) $$k_{0}=k_{a}\cdot {\left[\mathrm{ADP}\right]}_{\mathrm{ss}}-k_{b}\cdot \frac{k_{d}\cdot \left(C-{\left[\mathrm{ADP}\right]}_{\mathrm{ss}}\right)}{k_{s}\cdot {\left[\mathrm{ADP}\right]}_{\mathrm{ss}}}$$

It is assumed that cell function requires ADP concentration to be at a certain level (i.e. [*ADP*]_*ss*_) and that in the absence of the perturbation; the cell has adjusted *E*_*ss*_ and the rate constant *k*_0_ (and perhaps *k*_*d*_ and *k*_*s*_) to meet this requirement. We assume that the cell will do this also at different values of *k*_*b*_. The implication is that if different values of *k*_*b*_ are considered, *k*_*a*_ is also different as defined by (36).

Considering a perturbation of *k*_*d*_ from its steady state value, one finds for the time dependence of the variation in the enzyme level:

(37) $$\delta \dot{E}=k_{a}\cdot \delta \left[\mathrm{ADP}\right]-k_{b}\cdot \mathrm{\delta E}$$

and for the time dependence of the variation in the level of ADP:

(38) $$\begin{array}{l} \delta \overset{\cdot }{\left[\mathrm{ADP}\right]}=-\left(k_{s}\cdot E_{\mathrm{ss}}+k_{d}\right)\cdot \delta \left[\mathrm{ADP}\right]-k_{s}\cdot {\left[\mathrm{ADP}\right]}_{\mathrm{ss}}\cdot \mathrm{\delta E}\\ \;+\left(C-{\left[\mathrm{ADP}\right]}_{\mathrm{ss}}\right)\cdot \delta k_{d} \end{array}$$

By integrating the time dependence of the change in enzyme level *δE* in (37) into (38) one finds (18). By substituting the steady state condition of *E*_*ss*_ in (35),

(39) $$\begin{array}{l} \delta \overset{\cdot }{\left[\mathrm{ADP}\right]}=-k_{d}\cdot C\cdot \delta \ln\left[\mathrm{ADP}\right]-k_{s}\cdot {\left[\mathrm{ADP}\right]}_{\mathrm{ss}}\cdot \mathrm{\delta E}\\ \;+\left(C-{\left[\mathrm{ADP}\right]}_{\mathrm{ss}}\right)\cdot k_{d}\cdot \delta \ln k_{d} \end{array}$$

For that change in ADP level to be time independent, the integrand in the integral control term should equal zero around steady state, so that:

(40) $$\mathrm{\delta E}=\frac{k_{a}}{k_{b}}\cdot \delta \left[\mathrm{ADP}\right]$$

By using this expression to eliminate the change in enzyme level from (39) for the time dependence of the change in ADP level and set the latter to zero, the robustness coefficient in (21) can be obtained. Similarly, by using (40) to eliminate the change in ADP level from (39), the control of enzyme level (19) can be obtained.

## Competing interests

The authors declare that they have no competing interests.

## Authors’ contributions

FH and HVW jointly discussed and developed the main ideas of paper, and both authors wrote the manuscript. FH developed the hierarchical supply–demand theory, proposed the control engineering analysis of the regulatory systems, and implemented the simulation studies. HVW provided the MCA and HCA analysis, developed the first ATP metabolism example, identified the three terms in control analysis, and interpreted the results biochemically. VF contributed to the kinetic analysis of the end-product and initial-product modules. All authors read and approved the final manuscript and HVW rewrote the final version.
